# Metformin Protects Against Diabetes-Induced Cognitive Dysfunction by Inhibiting Mitochondrial Fission Protein DRP1

**DOI:** 10.3389/fphar.2022.832707

**Published:** 2022-03-22

**Authors:** Yan Hu, Yile Zhou, Yajie Yang, Haihong Tang, Yuan Si, Zhouyi Chen, Yi Shi, Hao Fang

**Affiliations:** ^1^ Department of Anesthesiology, Zhongshan Hospital, Fudan University, Shanghai, China; ^2^ Department of Anesthesiology, Jinshan Hospital, Fudan University, Shanghai, China; ^3^ Department of Anesthesiology, Minhang Branch, Zhongshan Hospital, Fudan University, Shanghai, China; ^4^ Institute of Clinical Science, Zhongshan Hospital, Fudan University, Shanghai, China; ^5^ Shanghai Key Laboratory of Organ Transplantation, Zhongshan Hospital, Fudan University, Shanghai, China

**Keywords:** diabetes, cognitive dysfunction, mitochondrial fission, dynamin-related protein 1, reactive oxidative stress, apoptosis

## Abstract

**Objectives:** Diabetes is an independent risk factor for dementia. Mitochondrial dysfunction is a critical player in diabetes and diabetic complications. The present study aimed to investigate the role of mitochondrial dynamic changes in diabetes-associated cognitive impairment.

**Methods:** Cognitive functions were examined by novel object recognition and T-maze tests. Mice hippocampi were collected for electron microscopy and immunofluorescence examination. Neuron cell line HT22 and primary hippocampal neurons were challenged with high glucose *in vitro*. Mitotracker-Red CM-H2X ROS was used to detect mitochondrial-derived free radicals.

**Results:** Diabetic mice exhibited memory loss and spatial disorientation. Electron microscopy revealed that diabetic mice had larger synaptic gaps, attenuated postsynaptic density and fewer dendritic spines in the hippocampus. More round-shape mitochondria were observed in hippocampal neurons in diabetic mice than those in control mice. In cultured neurons, high glucose induced a high phosphorylated level of dynamin-related protein 1 (DRP1) and increased oxidative stress, resulting in cell apoptosis. Inhibition of mitochondrial fission by Mdivi-1 and metformin significantly decreased oxidative stress and prevented cell apoptosis in cultured cells. Treatment of Mdivi-1 and metformin restored cognitive function in diabetic mice.

**Conclusion:** Metformin restores cognitive function by inhibiting mitochondrial fission, reducing mitochondrial-derived oxidative stress, and mitigating neuron loss in hippocampi of diabetic mice. The protective effects of metformin shed light on the therapeutic strategy of cognitive impairment.

## Introduction

The prevalence of diabetes has reached 11.1% in 2019 (Saeedi et al., 2019). Diabetic patients have a higher incidence of dementia than the non-diabetic population by 1.5 fold (Gudala et al., 2013; Koenig et al., 2017), indicating that diabetes is an important risk factor for cognitive impairment (Desmond et al., 1993). Diabetes-associated cognitive impairments are attributed to insulin resistance (Talbot et al., 2012; de la Monte, 2014), cerebral vascular endothelial dysfunction (De Silva and Faraci, 2016; Sheen and Sheu, 2016), enhanced oxidative stress (Yao-Wu Liu et al., 2016; González-Reyes et al., 2016), and increased neuron loss (Zuloaga et al., 2016).

*Hippocampus* is a brain structure, which is critical for learning, memory, and spatial discrimination. Alterations in the structure and function of hippocampal neurons have a significant impact on cognition. In hippocampal neurons of patients with Alzheimer’s (Fang et al., 2019; Oliver and Reddy, 2019) and Parkinson’s diseases (Yang et al., 2014; Ho et al., 2018), excessive mitochondrial fission has been reported. Mitochondrial fission, when balanced with fusion, maintains mitochondrial dynamics and is critical for mitochondrial hemostasis (Dorn and Kitsis, 2015; Yang et al., 2015). Excessive fission results in mitochondrial fragmentation: the fragmented mitochondria are cleared by mitophagy (Youle and Narendra, 2011) and autophagy (Barsoum et al., 2006; Twig et al., 2008); however, when the damaged mitochondria are not effectively cleared, their accumulation in cells results in increased oxidative stress (Sanderson et al., 2013) and cell apoptosis (Fossati et al., 2016; Xu et al., 2016). Mitochondrial fission is regulated by dynamin-related protein 1 (DRP1) (Youle and Narendra, 2011). In neurons subjected to oxygen/glucose deprivation-induced neuroexcitatory toxicity, the protein expression and/or phosphorylation at serine 616 residue (an activation site (Youle and Narendra, 2011)) of DRP1 is increased (Zhao et al., 2013; Flippo and Strack, 2017; Fang et al., 2019). The importance of DRP1 in mitochondrial structure and cell function is further confirmed by the findings that overexpressing DRP1 protein in embryonic hippocampal neurons alters mitochondrial structures and impairs dendritic branch formation (Dickey and Strack, 2011), and that inhibiting DRP1 protein restores mitochondrial density, increases ATP generations (Huang et al., 2015), prevents mitochondrial membrane potential loss (Frank et al., 2001), and protects neurons from ischemic stroke (Flippo et al., 2018).

Metformin is a first-line pharmacological treatment of diabetes by controlling postprandial blood glucose levels, downregulating glycogen synthesis, and increasing insulin sensitivity (Rena et al., 2017). It is reported that metformin treatment improves neuronal function in patients with diabetes (Campbell et al., 2018), neurodegenerative disease (Koenig et al., 2017), as well as in senile populations (Allard et al., 2016; Valencia et al., 2017; Samaras et al., 2020). The protective mechanisms of metformin include activation of adenosine 5′monophosphate-activated protein kinase (AMPK) (Frank et al., 2001; Zifeng Liu et al., 2016; Syngelaki et al., 2016; Xin et al., 2016; Sam and Ehrmann, 2017; Salvatore et al., 2020; Yang et al., 2020), inhibition of DRP1 protein (Wang et al., 2017), and prevention of ROS-induced cell apoptosis (Bhatt et al., 2013). Therefore, the present study was aimed to determine the effects of diabetes on mitochondrial dynamic changes in hippocampal neurons and the outcome on cognitive function of mice. Moreover, the effects, and the underlying mechanism, of metformin on hippocampal neuronal mitochondrial dynamic were investigated.

## Materials and Methods

### Animal Administration

Type 2 diabetic mice, db^-/-^ mice, and their genetic control m/m mice were bought from Cavens Laboratory Animal Corporation (Zhejiang, China). Eighteen six-week-old db^-/-^ and 6 m/m control male mice were housed in Zhongshan animal facility with 12-h light-dark cycles. All mice had free access to drinking water and regular chow. Metformin (≈250 mg/kg/d, Sigma-Aldrich, MO) was added in drinking water since week six (Sun et al., 2018). Mdivi-1 (1.2 mg/kg/d, Sigma-Aldrich, MO), a specific inhibitor of DRP1, was administered by a subcutaneous micro-osmotic pump (Alzet, Braintree, MA) since week eight (Wang et al., 2017). In week ten, mice were fasted and sacrificed by sodium pentobarbital injection (100 mg/kg i. p Sinopharm, Shanghai, China).

The newborn mice were purchased from Jiesijie Corporation (Shanghai, China) for primary neuron isolation.

The Animal Ethics Committee of Zhongshan Hospital Fudan University approved the study protocols.

### Novel Object Recognition Test

A novel object recognition test was applied to evaluate mice memory (Guimarães et al., 2017). The experiment was carried out in a 40*40*40 cm box. Before the experiment, mice were accustomed to two identical objects for 10 minutes in the chamber. Short-term memory was examined in 1 hour, and long-term memory was examined in 24 hours. In the experiment, one of the two identical objects was replaced by a novel subject. Mice movement in the chamber was recorded. A discrimination index was applied to examine their preference for the new object.

$$\mathrm{Discrimination}\;\mathrm{index}=\frac{\text{times}\;\text{explored}\;\text{new}\;\text{object}-\text{times}\;\text{explored}\;\text{old}\;\text{object}}{\text{times}\;\text{explored}\;\text{new}\;\text{object}+\text{times}\;\text{explored}\;\text{old}\;\text{object}}\times 100\text{\%}$$

### Spontaneous Alternation in T-Maze

Spontaneous alternation was examined in a T-maze (Ragozzino et al., 1996; Maynard et al., 2020). Mice were accustomed to the maze for 10 minutes. A successful entry was counted when a mouse entered an arm of the maze. Mice were credited when they entered the three different arms in three consecutive entries, not credited when they discontinuously chose one arm and credited minus when they repeatedly entered the same arm in three consecutive entries. The final scores were calculated for 5 minutes or a total of 15 entries (Supplementary Figure 1B).

### Cell Culture

The HT22 hippocampal neuronal cells (JennioBiotech, Guangzhou, China) were cultured in 30 mm dishes with Dulbecco Essential Medium (DMEM, Hyclone, Logan, United States), including 10% fetal bovine serum (FBS, Zhong Qiao Xin Zhou Biotech, Shanghai) and 1% penicillin-streptomycin (Thermo Fisher Scientific, United States).

### Primary Hippocampal Neuron Culture

Primary hippocampal neurons were isolated from newborn hippocampi with accutase (#A11105-01, Life Technologies, CA). After filtering through a 40 µm strainer (Falcom, United States), 4 × 10^^5^ neurons were seeded in poly-d-lysine (Sigma-Aldrich, MO)-coated dishes and cultured with DMEM medium containing 10% FBS and 10% horse serum (Solarbio Life Sciences, Beijing, China). Six hours later, neurobasal medium (#10888022, Life Technologies, CA) containing B27 supplement (#17504, Life Technologies, CA), 0.5 mM glutamine (#35050, Life Technologies, CA), and cytosine arabinoside (1 ug/mL, # C3350000, Sigma-Aldrich, MO) was added. Culture medium was changed every 2 days. Experiments were performed on day seven. The primary hippocampal neurons were positively stained with NeuN (#ab104224, Abcam, UK), a neuron-specific marker (Supplementary Figure 1C).

### Western Blotting

Total protein of cultured cells was prepared with lysis buffer (150 mmol/L NaCl, 1 mmol/L EDTA, 1 mmol/L NaF, 1 mmol/L dithiothreitol, 10 μg/μl aprotinin, 10 μg/μl leupeptin, 0.1 mmol/L Na_3_VO_4_, 1 mmol/L phenylmethylsulfonyl fluoride, and 0.5% NP-40). Protein extracts (20 μg) were loaded in 10–12.5% sodium dodecyl sulfate-polyacrylamide gel for electrophoresis. Proteins were transferred to 0.45 µm polyvinylidene fluoride membranes (Merck Millipore, Darmstadt, Germany). After blocking with 5% non-fat milk/Tris-buffered saline containing 0.1% Tween-20 at room temperature for 1 hour, the membranes were incubated with primary antibodies at 4°C overnight. Then the membranes were washed and incubated with secondary antibodies (1:1,000) for 1 hour at 37°C. After washing, protein expression levels were normalized to β-actin with ImageJ (NIH, Bethesda, MD).

### Mitochondrial-Derived Oxidation Product Detection

Mitotracker-Red CM-H2X ROS (#M7513, Thermo Fisher, CA) was used to evaluate mitochondrial-derived oxygen-derived free radicals. After 6-h high glucose stimulation, neurons were incubated with Mitotracker-Red CM-H2X ROS solution (1 µM) at 37°C for 15 minutes and Hoechst 33342 (#C1029, Beyotime Biotechnology, Shanghai, China) for 5 minutes.

Brain samples were fixed with 4% paraformaldehyde, blocked in 5% goat serum (Absin Biomart, Shanghai, China) at 37°C for 20 minutes. The samples were incubated with dihydroethidium (10 μM, Beyotime Biotechnology, Shanghai, China) for 1 hour. Hoechst 33342 (#C1029, Beyotime Biotechnology, Shanghai, China) for 5 minutes. Fluorescent signals in hippocampal CA1 region were detected using a fluorescence microscope (Olympus, Tokyo, Japan) and analyzed with ImageJ (NIH, Bethesda, MD).

### TUNEL Assay

Cells and brain slices were fixed with 4% paraformaldehyde, then incubated with One-Step-TUNEL apoptosis kit (Beyotime Biotechnology, Shanghai, China) at 37 °C for 1 hour. DAPI (#C1002, Beyotime Biotechnology, Shanghai, China) for 5 minutes. TUNEL signals in the hippocampal CA1 region were detected by a fluorescence microscope and analyzed with ImageJ.

### Tissue Preparation and Immunofluorescence Examination

Mice brains were dehydrated with 30% sucrose solution and embedded in a tissue freezing medium (OCT, SAKURA Tissue-Tek, CA, United States). The frozen samples were sectioned for 5 µm thickness and fixed with 50% ethanol, including 5% glacial acetic acid and 5% formaldehyde. After washing in phosphate buffer saline (Sangon Biotech, Shanghai, China), brain samples were blocked in 5% goat serum at 37°C for 20 minutes. The samples were incubated with primary antibodies [p-DRP1 (1:800, #4494, Cell Signaling Technology, Boston, MA), DRP1 (1:50, #8570, Cell Signaling Technology, Boston, MA), cleaved-caspase 3 (1:400, #9661, Cell Signaling Technology, Boston, MA)], Dihydroethidium (DHE) and NeuN at 4°C overnight. Tissues were incubated with secondary antibodies (1:200, #A1034, Thermo Fisher Scientific, CA; 1:200, #115–585–003, Jackson, MI) at 37°C for 1 hour. Images were obtained using a fluorescence microscope (Olympus, Tokyo, Japan).

### Transmission Electron Microscope (TEM)

Mice brains were fixed with 4% paraformaldehyde with 1% glutaraldehyde. Hippocampal neurons, including their mitochondria synapses (Supplementary Figure 1D) and dendritic spines, were recorded. Neuron mitochondria, synaptic gaps, postsynaptic density (PSD), and dendritic spines were measured blindly by lab technicians.

The short axis and the long axis, which is perpendicular to each other, were measured for the ratio of the short-axis/long axis. The image was enlarged enough to measure the pixels of synaptic gap and postsynaptic density and calculated according to the pixels corresponding to the ruler. Three points were selected from each synapse and the average value was taken as the data for statistical analysis. These measured values agree with those reported in the literature (Shields et al., 2015). Select the dendrite structure as complete as possible, observe the synaptic structure formed between it and surrounding structures, including axons and dendrites, and count it as the close connection between dendrites and other structures.

## Materials

Hippocampal neuronal cell line HT22 was purchased from Jennio Biotech Corporation (Biotech, Guangzhou, China). Mitochondrial Dynamics Antibody Sampler Kit, *p*-AMPK, AMPK, caspase 3, cleaved caspase 3, ATG5, ATG7, β-actin purchased from Cell Signaling Technology (CST, Boston, MA), while Pink1 antibody was purchased from Novus Biologicals (Colorado, CO). Horseradish peroxidase (HRP)-conjugated goat anti-mouse and goat anti-rabbit secondary antibodies were purchased from Jackson Corporation (Missouri, MO). Metformin (Metformin hydrochloride), D-mannitol, mitochondrial division inhibitor 1 (Mdivi-1), and GSK 621 were obtained from Sigma Chemical (St. Louis, MO). Compound C (dorsomorphin dihydrochloride) was purchased from MedChemExpress (New Jersey, NJ). Fetal bovine serum (FBS) was obtained from Hyclone (Logan, UT). Living cell dye Mitotracker-Red CM-H2X ROS was obtained from Thermo Fisher Scientific (California, CA), Hoechst was purchased from Beyotime Biotechnology (Shanghai, China). Antibiotic (penicillin, streptomycin) was purchased from Thermo Fisher Scientific (California, CA). Subcutaneous micro-osmotic pumps were purchased from Durect Corporation (California, CA). Glucophage (metformin sustained-release tablets) was purchased from Sino-American Shanghai Squibb Pharmaceutical Corporation (Shanghai, China). Sodium pentobarbital is bought from Sinopharm Chemical Reagent Corporation (Shanghai, China).

### Statistical Analysis

All quantitative data were expressed as means ± SEM and were analyzed by one-way analysis of variance (ANOVA) or Student’s test, as appropriate. A *p*-value less than 0.05 was considered statistically significant. All statistical analyses were performed with GraphPad Prism 7.04 (GraphPad, San Diego, CA).

## Results

### Diabetic Mice Exhibit Cognitive Dysfunction

**FIGURE 1:**
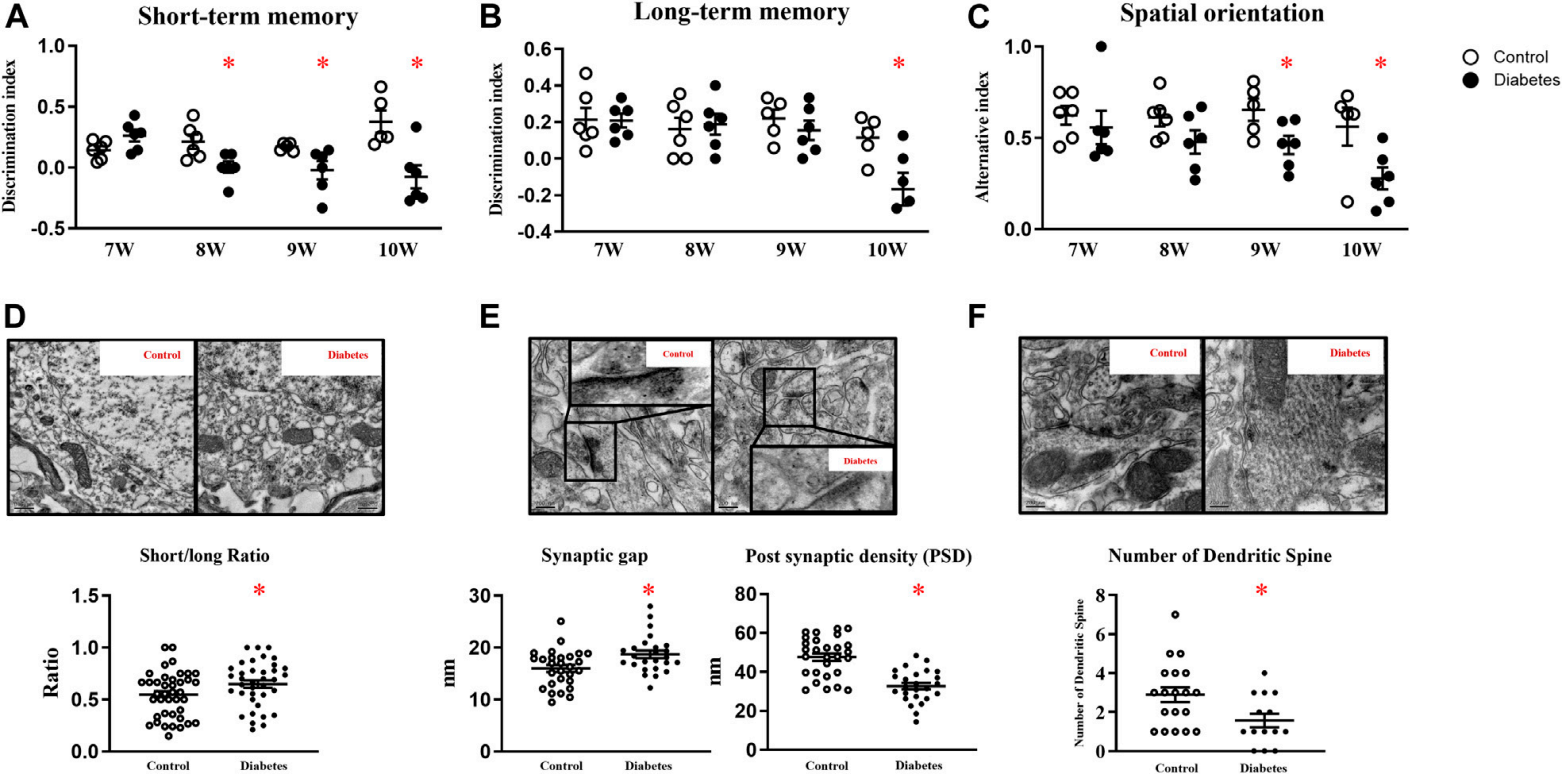
Diabetic mice exhibit cognitive deficits. Diabetic mice exhibit lower scores in short-term memory **(A)**, long-term memory **(B)**, and spatial orientation tests **(C)**. **(D)** Representative picture of mitochondria in hippocampal neurons (upper panel) and the ratio of short/long axis of mitochondria (40 mitochondria from control, 37 from diabetic mice, *n* = 3) (200,00 ×, Bar = 0.5 µm) (lower panel). **(E)** Representative picture of a synaptic gap (upper panel) in the hippocampus and measurement of synaptic gaps and PSD (lower panel) (28 synapses from control, 25 from diabetic mice, *n* = 3). (500,00 ×, Bar = 200 nm) **(F)** Representative picture of dendritic spines in the hippocampus (upper panel) and numbers of dendritic spines per dendrite (lower panel) (19 dendrites from control, 14 from diabetic mice, *n* = 3) (500,00 ×, Bar = 200 nm) Data presented as means ± SEM, *p < 0.05 vs control.

Cognitive functions were examined when mice were seven-week-old, since db^-/-^ mice had consistently high glucose levels at week six. In week seven, control and diabetic mice had comparable scores in memory and spatial orientation tests. Compared with age-matched control mice, diabetic mice exhibited lower scores in short-term memory tests since week eight and in spatial orientation tests since week nine. In week ten, long-term memory was declined in diabetic mice as well (Figures 1A–C; Supplementary Figure 1A).

TEM revealed that control mice had slender mitochondria with clear cristae, while diabetic mice had swelling and round-shape mitochondria with some vacuolation. Mitochondrial short axis to its long axis ratio was significantly increased in diabetic mice (Figure 1D). Compared with control mice, synaptic gaps in diabetic mice were larger (control vs diabetes: 16 ± 0.67 nm: 18.69 ± 0.72 nm, 28 from 3 control and 25 from 3 diabetic mice, *p* < 0.05), and PSD in diabetic mice was significantly attenuated (control vs diabetes: 47.68 ± 1.93 nm: 32.76 ± 1.68 nm, 28 from 3 control and 25 from 3 diabetic mice, *p* < 0.05, Figure 1E) In line, diabetic mice had fewer dendritic spines in hippocampal neurons than control m/m mice (control vs diabetes: 2.89 ± 1.66: 1.57 ± 1.57 per dendrite, 19 from 3 control and 14 from 3 diabetic mice, *p* < 0.05, Figure 1F).

### High Glucose Increases Neuron Mitochondrial Fission and Induces Neuron Apoptosis

**FIGURE 2:**
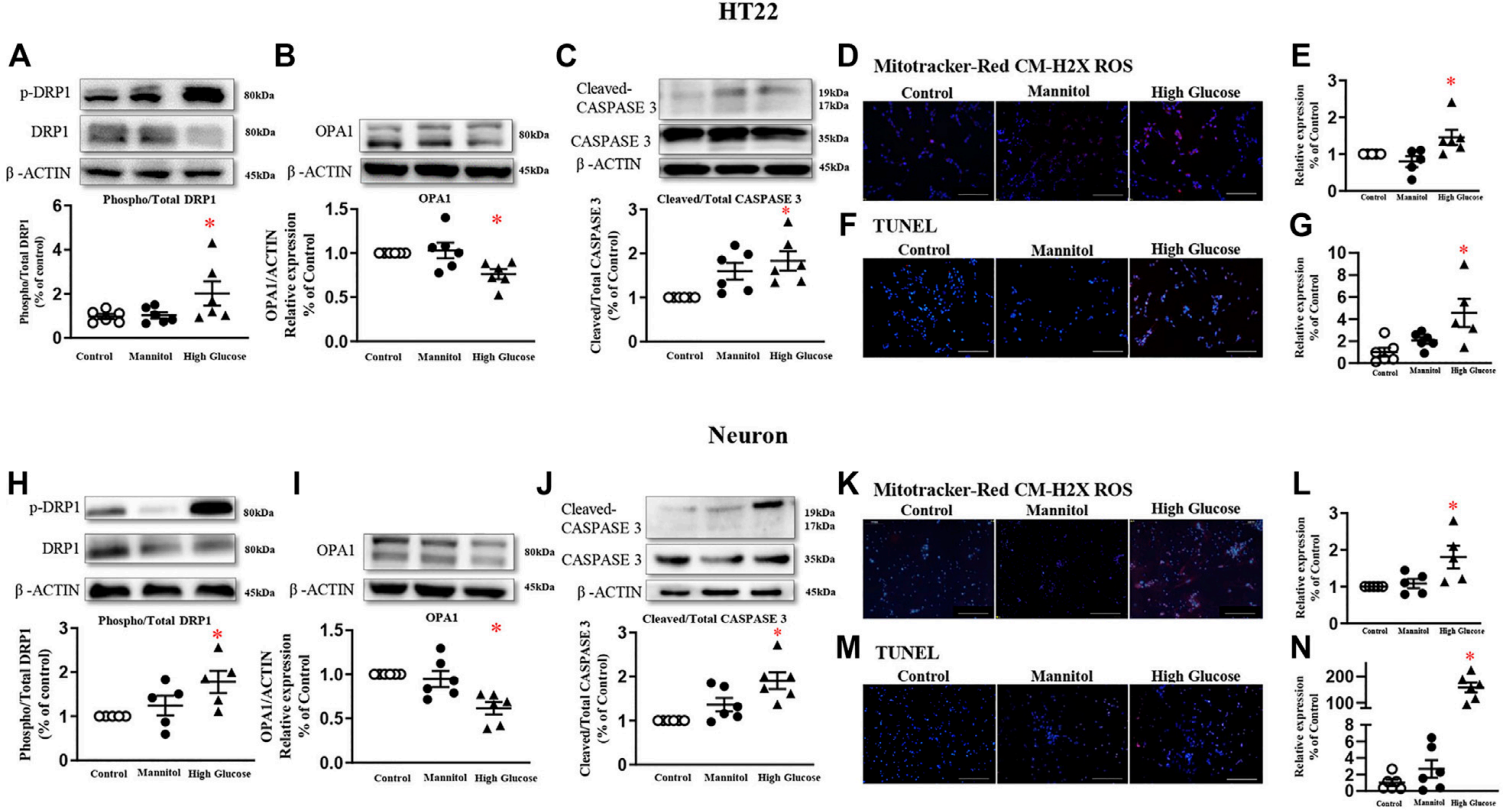
High glucose increases mitochondrial fission in neurons and induces neuron apoptosis in HT22 (A-G, upper panel) and neurons (H-N, lower panel). Representative blots and densitometric quantification of DRP1, OPA1, and caspase proteins in HT22 **(A–C)** and neurons **(H–J)**. The phosphorylation of DRP1 (top blots) was normalized to total DRP1 protein (middle), and total DRP1 was normalized to β-ACTIN (bottom). Fluorescent signals and quantification of Mitotracker-Red CM-H2X ROS in HT22 **(D–E)** and primary neurons **(K–L)** stimulated with high glucose. (200 ×, Bar = 800 µm). Hoechst labeled the nuclei (blue), and Mitotracker-Red CM-H2X ROS was stained in red (Fluor 594). Fluorescent signals and quantification of TUNEL in HT22 **(F–G)** and primary neurons **(M–N)** stimulated with high glucose. (200 ×, Bar = 800 µm). DAPI labeled the nuclei (blue), and TUNEL was stained in red (Fluor 594). Data presented as means ± SEM, *p < 0.05 vs cells under control condition by One-way ANOVA.

In HT22 cells, a cell line immortalized from primary mouse hippocampal neuronal, 6-h high glucose stimulation induced a higher phosphorylated level of DRP1 at serine 616 residue in a dose-dependent manner, while the total protein expression was comparable (Figure 2A, and Supplementary Figure 2). Mannitol, the osmotic control, did not increase the phosphorylated or total level of DRP1 protein (Figure 2A, and Supplementary Figure 3A). Therefore, a 60 mmol/L glucose stimulation was used in the present study. High glucose significantly reduced protein expression of mitochondrial fusion protein optic atrophy 1 (OPA1, Figure 2B), but did not affect protein expression of mitochondrial fission protein MFF (Supplementary Figure 3B), the receptor of DRP1. High glucose failed to change protein expressions of Pink1, autophagy-related 5 (Atg5), or Atg7 (Supplementary Figures 3C–D).

Six-hour high glucose stimulation significantly increased the phosphorylated level of DRP1 (Figure 2H), but not the total protein expression in primary neurons (Supplementary Figure 4A). High glucose stimulation significantly reduced the protein expression of OPA1 (Figure 2I). High glucose did not affect protein expressions of MFF, Pink1, Atg5, or Atg7 (Supplementary Figures 4B–D).

Mitochondrial-derived oxidative stress, detected by Mitotracker-Red CM-H2X ROS, was significantly increased in both HT22 (Figures 2D,E) and primary neurons stimulated with high glucose (Figure 2K-L).

After 24 h, high glucose increased cleaved-caspase 3 protein expression, but not the total protein, in both HT22 (Figure 2C) and primary hippocampal neurons (Figure 2J). TUNEL assay detected more fluorescent signals in cultured cells stimulated with high glucose (Figures 2F,G, M-N).

### Inhibition of Mitochondrial Fission Decreases Oxidative Stress and Reduces Apoptosis in Cultured Neurons

To explore the role of DRP1 in high glucose-induced mitochondrial dysfunction, Mdivi-1 (25 µM) (Cui et al., 2016) and metformin (10 mM) (Meng et al., 2016) were used in the present study.

**FIGURE 3:**
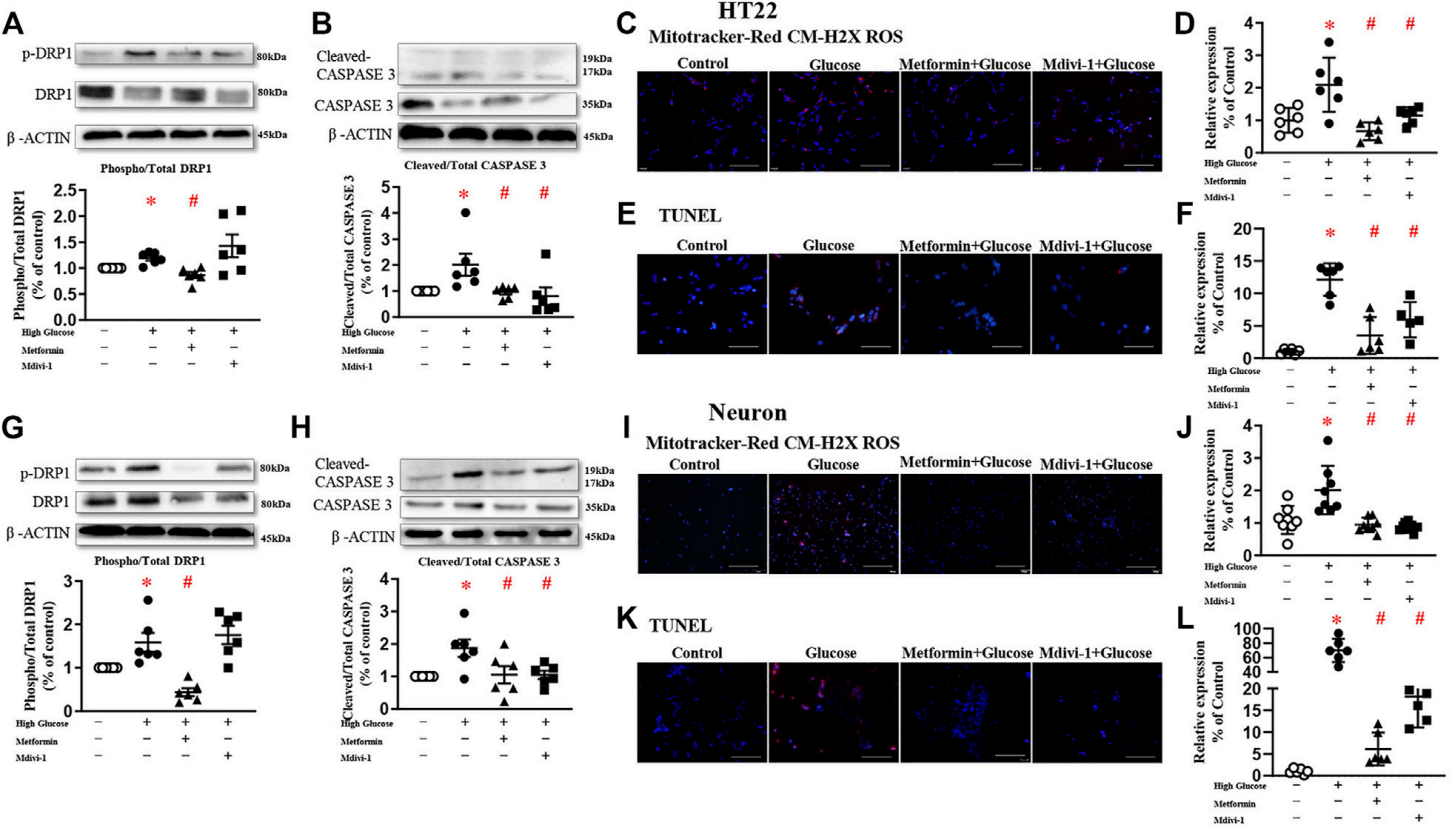
Inhibition of mitochondrial fission decreases oxidative stress and reduces apoptosis in HT22 (**(A–F)**, upper panel) and neurons (**(G–L)**, lower panel). Representative blots and densitometric quantification of phosphorylated DRP1 and caspase 3 in HT22 **(A–B)** and neurons **(G–H)**. The phosphorylation of DRP1 (top blots) was normalized to total DRP1 protein (middle), and total DRP1 was normalized to β-ACTIN (bottom). The cleaved of caspase3 (top blots) was normalized to caspase3 protein (middle), and caspase3 was normalized to β-ACTIN (bottom). Fluorescent signals and quantification of Mitotracker-Red CM-H2X ROS in HT22 **(C–D)** and primary neurons **(I–J)** stimulated with high glucose (200 ×, Bar = 800 µm). Fluorescent TUNEL signals and quantification in HT22 **(E–F)** and primary neurons **(K–L)** stimulated with high glucose. (200 ×, Bar = 800 µm). DAPI labeled the nuclei (blue), and TUNEL was stained in red (Fluor 594). Data presented as means ± SEM, *p < 0.05 vs cells under control condition, #*p* < 0.05 vs cells stimulated with high glucose by One-way ANOVA.

Mdivi-1 incubation did not affect DRP1 protein expressions. Incubation of metformin significantly reduced the phosphorylated level of DRP1 in both HT22 and primary hippocampal neurons. Mdivi-1 and metformin alone significantly decreased high glucose-induced oxidative levels in HT22 and primary hippocampal neurons. Mdivi-1 and metformin incubation significantly reduced cleaved-caspase 3 expressions and the TUNEL signals in HT22 and primary hippocampal neurons (Figure 3).

### The Protective Effect of Metformin Is Attributed to the Reduced Phosphorylation of DRP1 but Not AMPK Activation

A large body of literature has reported that metformin exerts its protective effect through AMPK-dependent (Rena et al., 2017; Salvatore et al., 2020) or -independent mechanisms (Cui et al., 2016; Chan et al., 2020; Silva et al., 2021). To study the involvement of AMPK in DRP1 activation, AMPK agonist GSK 621 (Jiang et al., 2016) was used in the absence of metformin, while AMPK antagonist Compound C (Liu et al., 2014) was incubated in the presence of metformin.

**FIGURE 4:**
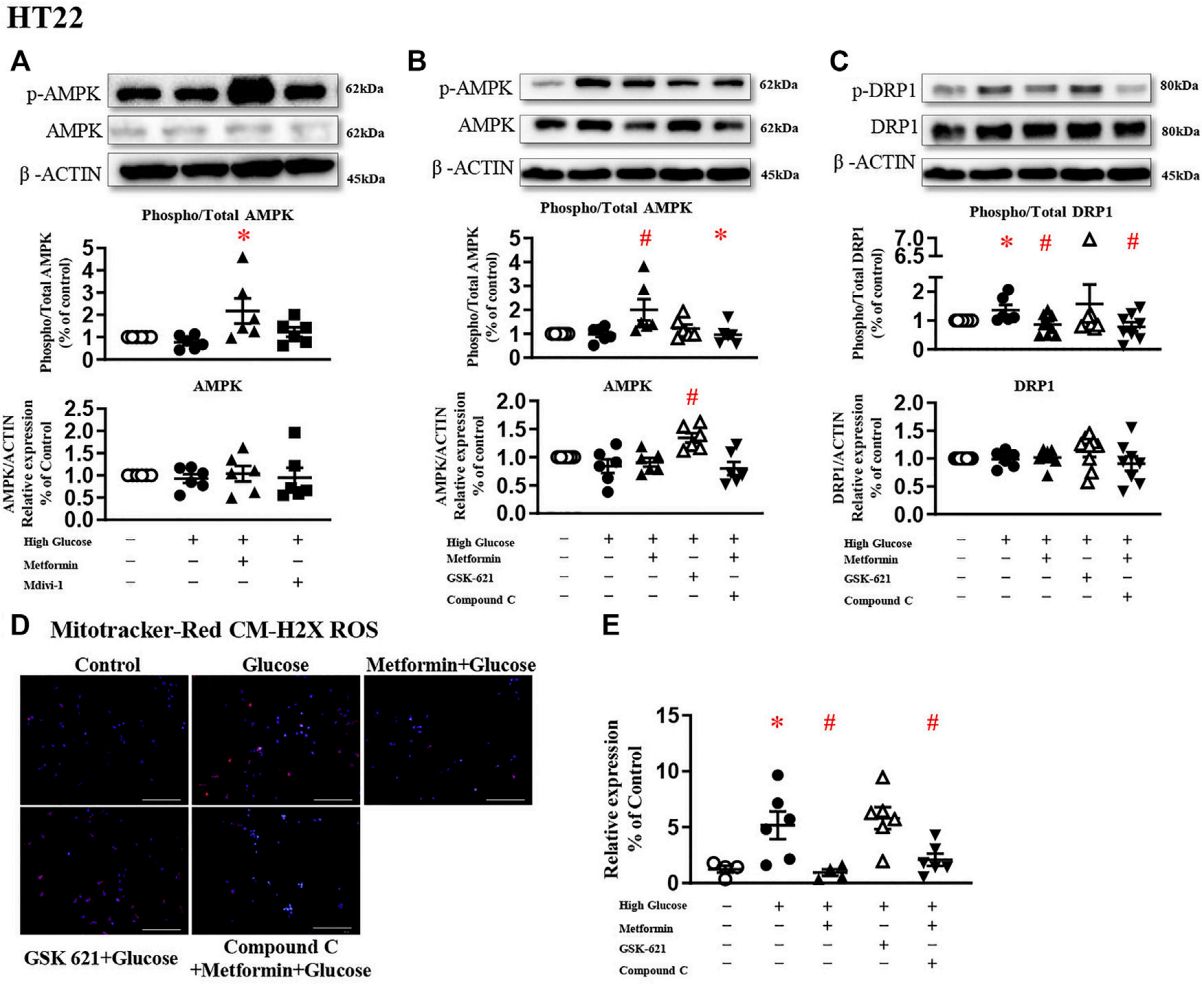
The protective effect of metformin is attributed to DRP1 phosphorylation but not AMPK activation in HT22. **(A)** Representative blots and densitometric quantification of AMPK protein in HT22 stimulated high glucose in the treatment of metformin or Mdivi-1. The phosphorylation of AMPK (top blots) was normalized to total AMPK protein (middle), and total AMPK was normalized to β-ACTIN (bottom). **(B)** Representative blots and densitometric quantification of AMPK protein in HT22 treated with AMPK agonist GSK 621 in the absence of metformin, or AMPK antagonist Compound C in the presence of metformin. The phosphorylation of AMPK (top blots) was normalized to total AMPK protein (middle), and total AMPK was normalized to β-ACTIN (bottom). **(C)** Representative blots and densitometric quantification of DRP1 protein in HT22 AMPK agonist GSK 621 in the absence of metformin, or AMPK antagonist Compound C in the presence of metformin. The phosphorylation of DRP1 (top blots) was normalized to total DRP1 protein (middle), and total DRP1 was normalized to β-ACTIN (bottom). Fluorescent signals of Mitotracker-Red CM-H2X ROS **(D)** (200 ×, Bar = 800 µm) and quantification **(E)** in HT22 protein in HT22 AMPK agonist GSK 621 in the absence of metformin, or AMPK antagonist Compound C in the presence of metformin. Data presented as means ± SEM, *p < 0.05 vs cells under control condition, #*p* < 0.05 vs cells stimulated with high glucose by One-way ANOVA.

In the present study, metformin significantly increased AMPK phosphorylation in HT22 challenged with high glucose (Figure 4A). In the absence of metformin, GSK 621 increased the AMPK expression (Figure 4B), but failed to reduce the DRP1 expressions (Figure 4C). GSK 621 did not prevent high glucose-induced oxidative stress (Figures 4D,E). In the presence of metformin, Compound C significantly downregulated the phosphorylation of AMPK (Figure 4B) and DRP1 (Figure 4C), and diminished high glucose-induced oxidative stress (Figures 4D,E).

**FIGURE 5:**
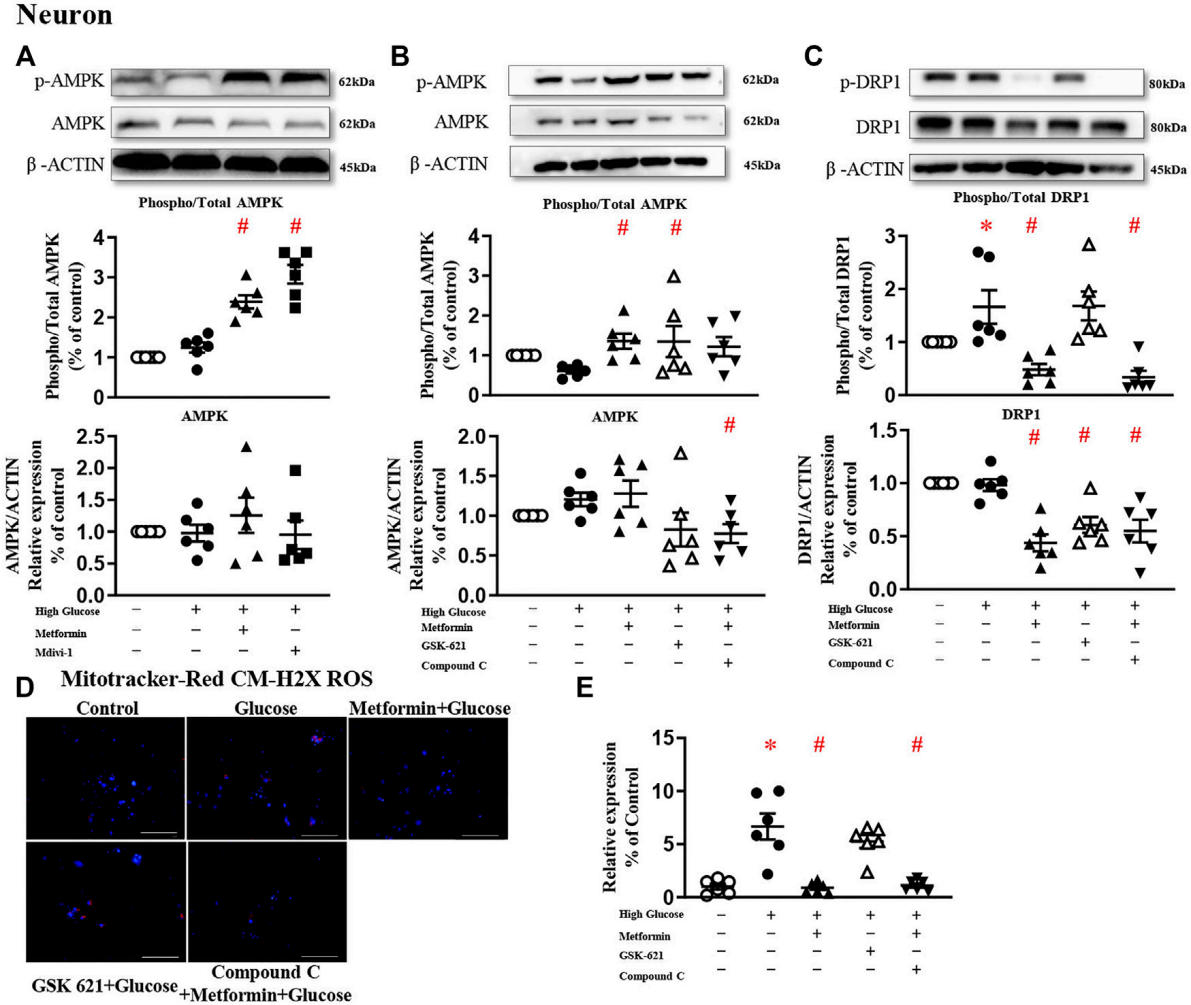
The protective effect of metformin is attributed to DRP1 phosphorylation but not AMPK activation in primary neurons. **(A)** Representative blots and densitometric quantification of AMPK protein in primary neurons. The phosphorylation of AMPK (top blots) was normalized to total AMPK protein (middle), and total AMPK was normalized to β-ACTIN (bottom). **(B)** Representative blots and densitometric quantification of AMPK protein in primary neurons stimulated high glucose in the presence of GSK 621 or Compound C. The phosphorylation of AMPK (top blots) was normalized to total AMPK protein (middle), and total AMPK was normalized to β-ACTIN (bottom). **(C)** Representative blots, and densitometric quantification of DRP1 protein in primary neurons stimulated high glucose in the presence of GSK 621, or Compound C. The phosphorylation of DRP1 (top blots) was normalized to total DRP1 protein (middle), and total DRP1 was normalized to β-ACTIN (bottom). Fluorescent signals of Mitotracker-Red CM-H2X ROS **(D)** (200 ×, Bar = 800 µm) and quantification **(E)** in primary neurons stimulated with high glucose in the presence of GSK 621, or Compound C. Data presented as means ± SEM, *p < 0.05 vs cells under control condition, #*p* < 0.05 vs cells stimulated with high glucose by One-way ANOVA.

Metformin significantly increased AMPK phosphorylation in primary neurons challenged with high glucose (Figure 5A). In the presence of metformin, Compound C significantly downregulated AMPK protein expression, but did not change the phosphorylated level (Figure 5B). Compound C significantly reduced both phosphorylated and total protein expressions of DRP1 (Figure 5C), and diminished high glucose-induced oxidative stress (Figures 5D,E). In the absence of metformin, GSK 621 activated AMPK phosphorylation and reduced the total protein expression, but did not change the phosphorylated level of DRP1 (Figure 5C). Consistently, GSK 621 did not inhibit high glucose-induced oxidative stress (Figures 5D,E).

### Metformin Protects Against Cognitive Dysfunction in Diabetic by Inhibiting DRP1 Phosphorylation in the Hippocampus

To confirm the protective effects of mitochondrial fission inhibition on neuron function, diabetic mice were treated with metformin or Mdivi-1.

Treatment of metformin or Mdivi-1 did not significantly reduce serum glucose levels or body weight (Table 1).

**TABLE 1:** General conditions in diabetic mice.

	m/m control (*n* = 6)	db^-/-^ mice (*n* = 8)	Metformin treated db^-/-^ mice (*n* = 7)	Mdiv1 treated db^-/-^ mice (*n* = 6)
Weight (g)	21.63 ± 0.48	46.58 ± 1.04*	45.6 ± 0.58*	46.48 ± 0.51*
Blood glucose (mmol/L)	5.57 ± 1.16	22.03 ± 1.04*	18.67 ± 1.83*	21.83 ± 3.01*

Data shown as the means ± SEM, *p < 0.05 vs m/m control group.

**FIGURE 6:**
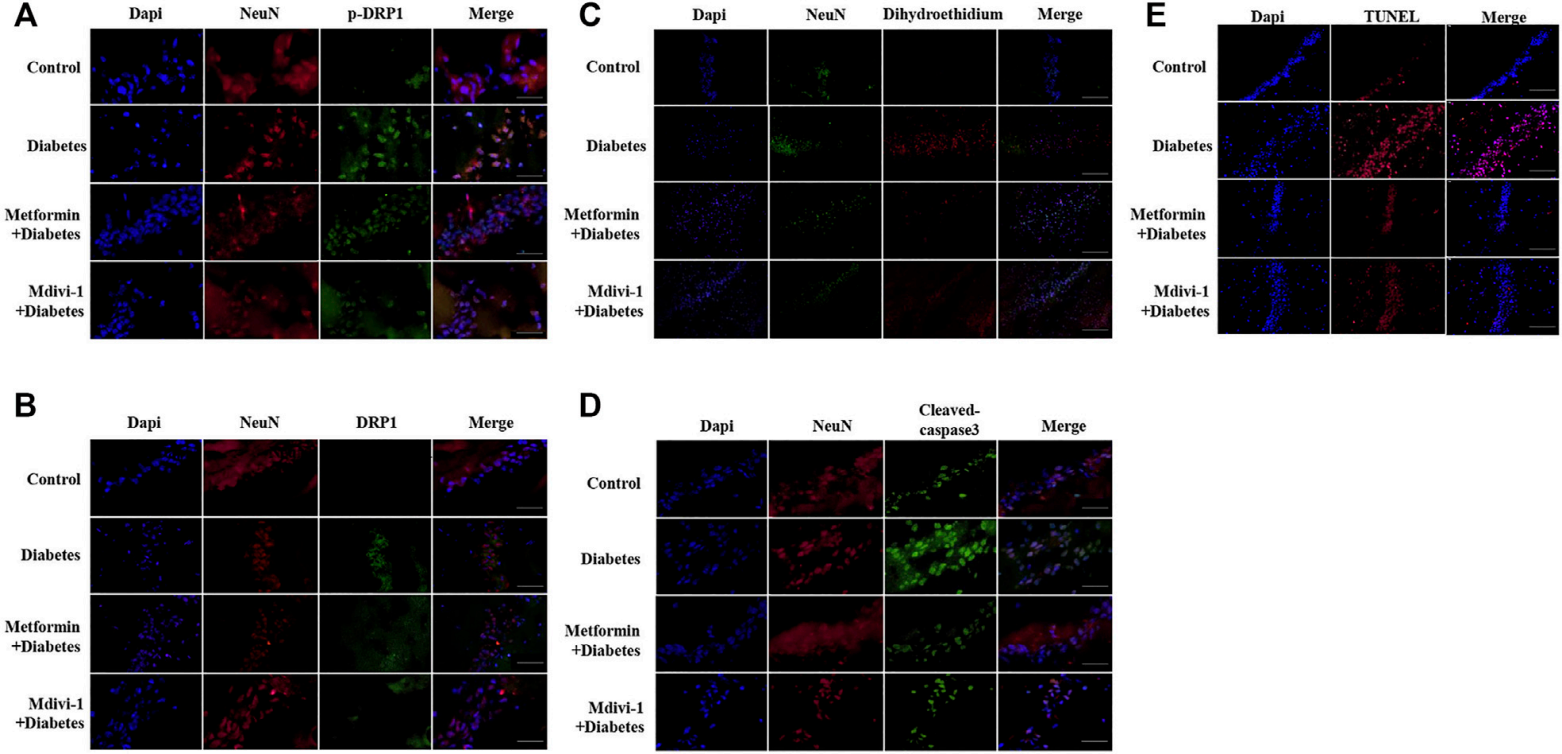
Metformin protects against cognitive dysfunction in diabetes by inhibiting DRP1 phosphorylation in the hippocampus. Presences of phosphorylated DRP1 **(A)** (400 ×) and total DRP1 protein **(B)** (400 ×) in hippocampal neurons. **(C)** Presences of oxidative products in hippocampal neurons (200×). **(D)** Presences of cleaved caspase-3 in hippocampal neurons (400×). **(E)** Presences of TUNEL signals in hippocampal neurons (200×) DAPI labeled the nuclei (blue), and TUNEL was stained in red (Fluor 594).

Presences of phosphorylated and total DRP1 protein were significantly increased in hippocampal neurons in db^-/-^ mice, compared with m/m mice. Metformin or Mdivi-1 significantly reduced phosphorylated and total protein of DRP1 protein, in hippocampal neurons (Figures 6A,B).

Signals of oxidative stress were significantly increased in hippocampal tissue in db^-/-^ mice. Metformin or Mdivi-1 administration significantly reduced the signals (Figure 6C).

In line with cultured cells, diabetic mice had significantly higher fluorescent signals of cleaved caspase-3 protein (Figure 6D) and TUNEL (Figure 6E), which were suppressed by metformin or Mdivi-1 treatment.

**FIGURE 7:**
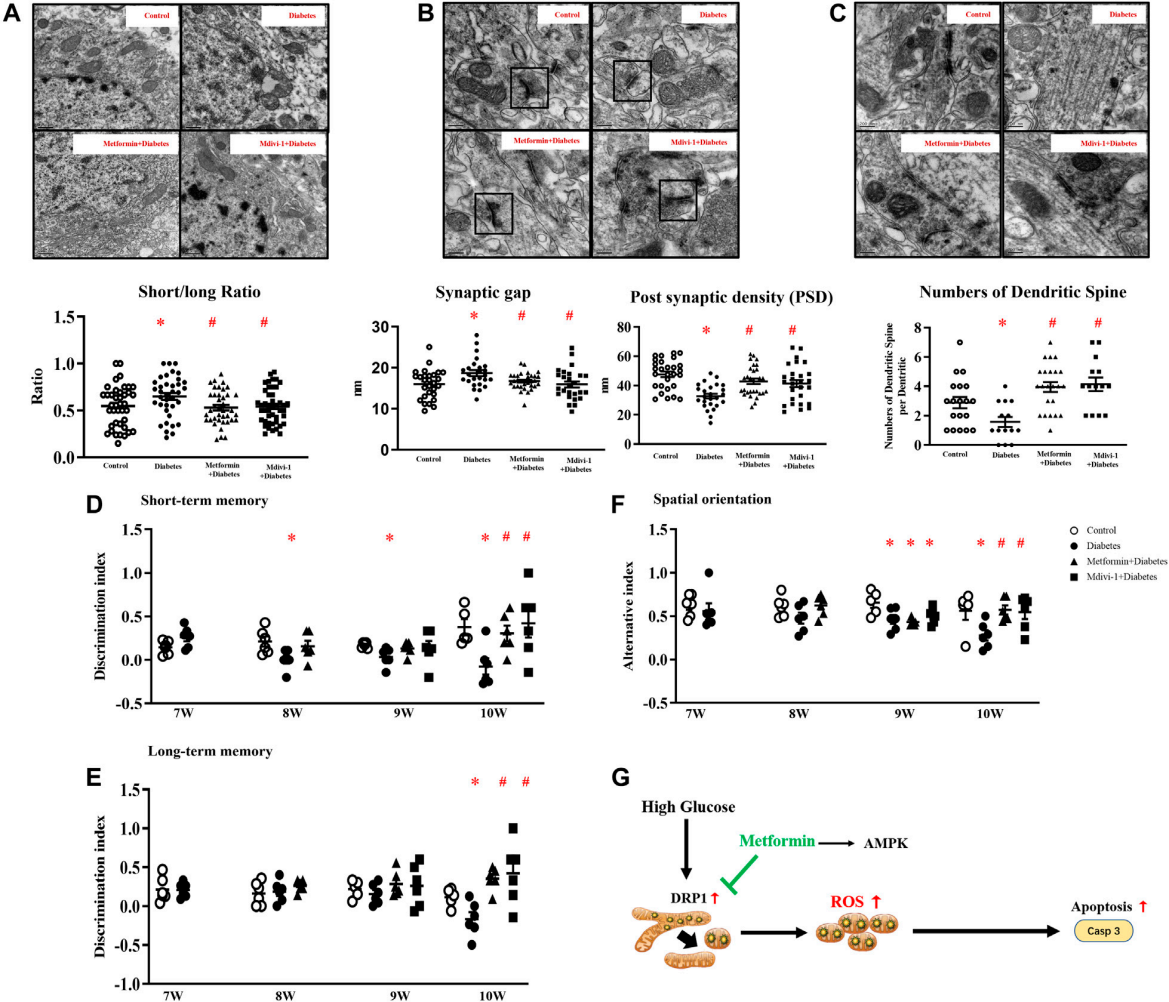
**(A)** Representative picture of mitochondria in hippocampal neurons (upper) and measurement of mitochondria short/long axis ratio (lower) (39 mitochondria from metformin treatment, 45 from Mdivi-1 treatment, *n* = 3, 200,00×, Bar = 0.5 µm) **(B)** Representative picture of a synaptic gap in the hippocampus (upper) and measurements of synaptic gap and PSD (lower) (30 synapses from metformin treatment, 26 from Mdivi-1 treatment, *n* = 3, 500,00×, Bar = 200 nm). **(C)** Representative picture of dendritic spines in the hippocampus (upper) and counts of dendritic spines per dendrite (lower) (23 dendrites from metformin treatment, 14 from Mdivi-1 treatment, *n* = 3, 500,00×, Bar = 200 nm). Metformin and Mdivi-1 protect against diabetes-induced cognitive dysfunction in short-term memory **(D)**, long-term memory **(E)**, and spatial disorientation **(F)**. Data presented as means ± SEM, *p < 0.05 vs m/m control, #*p* < 0.05 vs db^-/-^ control by One-way ANOVA **(G)** Schematic diagram. High glucose increases mitochondrial fragments by activating DRP1 protein, resulting in elevated oxidative stress and cell apoptosis. Metformin inhibits DRP1 phosphorylation, reduces mitochondrial-derived oxidative stress, and prevents neuron loss.

In addition, metformin or Mdivi-1 treatment restored mitochondria morphology (Figure 7A) and synapse structures and significantly increased dendritic spines (Figures 7B,C) in hippocampal neurons.

Mdivi-1 treatment restored diabetic mice cognitive function in memory tests and spatial orientation tests in week ten. Metformin significantly improved the short-term memory in diabetic mice since week eight and restored mice cognitive performance in spatial orientation and long-term memory tests in week ten (Figures 7D–F).

## Discussion

This study highlights the critical role of mitochondrial dynamics in the structure and function of hippocampal neurons. Diabetes/hyperglycemia alters mitochondrial dynamics in favor of fission, resulting in enhanced oxidative stress and apoptosis in neurons. Inhibiting mitochondrial fission reduces oxidative stress and protects mice from diabetes-induced cognitive impairment.

In this study, diabetic mice presented cognitive deficits with short-term memory loss, impaired spatial orientation, and declined long-term memory in chronological order, resembling the development of dementia in humans (Gale et al., 2018). These data provide scientific evidence that diabetes is an important risk for cognitive dysfunction (Desmond et al., 1993).

Mitochondria participate in ATP production, neurotransmitter synthesis, and cell apoptosis (Flippo and Strack, 2017; Flippo et al., 2018). An imbalance in mitochondrial dynamics, due to increases in fission and/or decreases in fusion, leads to mitochondrial fragmentation and oxidative stress (Shenouda et al., 2011; van der Bliek et al., 2013). In the present study, the hippocampal neurons of diabetic mice had swelling and round-shape mitochondria with disordered cristae, implying that the progression of diabetes is associated with abnormal mitochondrial dynamics in the hippocampal neurons. This, in turn, accounts for the neuronal dysfunction, as indicated by the increased synaptic gaps, attenuated PSD, fewer dendritic spines of the hippocampal neurons (Kamat et al., 2016), and impaired cognitive performance of diabetic mice.

The detrimental effect of diabetes on mitochondrial dynamic appears to be the consequence of elevated blood glucose level, since hippocampal neuronal cells incubated with high glucose concentration showed an increased activation of DRP1, the protein essential for the activation of mitochondrial fission (Fonseca et al., 2019). Activation of DRP1 is associated with pathological neurological conditions, such as brain tumor initiation (Xie et al., 2015), traumatic brain injury (Wu et al., 2016), and neurodegenerative diseases (Akhtar et al., 2016; Chuang et al., 2016). Inhibition of DRP1 preserves mitochondrial morphology and synaptic plasticity in the hippocampus of diabetic mice (Huang et al., 2015) and reduces microcystin-leucine-arginine-induced neuron apoptosis (Zhang et al., 2020). In agreement with these findings, treatment with diabetic mice with the DRP1 inhibitor Mdivi-1 prevents mitochondrial fission, inhibits neuron death, and restores cognitive function in diabetic mice, thus implying the critical role of DRP1 protein for neuronal functions. Nevertheless, serum glucose levels are comparable among diabetic groups, and the glucose levels are not correlated to mice cognitive performances (Supplementary figure 6). Of importance, metformin produces similar protective effects to those of Mdivi-1 against mitochondrial and cognitive dysfunction in diabetic mice. Therefore, the protective effects of metformin are attributed to the restoration of mitochondrial hemostasis (Wang et al., 2017) (Izzo et al., 2017), as confirmed by the finding that the phosphorylation and hence activation of DRP1 is decreased following metformin treatment.

Metformin exerts its beneficial pharmacological effects through AMPK-dependent (Mancini et al., 2017; Yang et al., 2020) and -independent (Cui et al., 2016; Meng et al., 2016) mechanisms. It is reported that AMPK is an upstream signal of mitochondrial fission since activation of AMPK inhibits mitochondrial fission and protects against energy stress in human osteosarcoma U2OS cells (Toyama et al., 2016) and lead-exposed human neuroblastoma SH-SY5Y cells (Yang et al., 2020). On the other hand, metformin protects retinal pigment epithelial cells against NaIO_3_ stress by stabilizing respiratory complex I, without activating AMPK (Meng et al., 2016). In the present study, DRP1 is not activated by the AMPK agonist GSK 621, thus suggesting that the protective effect of metformin against mitochondrial dysfunction in hippocampal neurons is through an AMPK-independent mechanism (Cui et al., 2016; Meng et al., 2016; Silva et al., 2021).

The mitochondrial fragments resulted from increased mitochondrial fission can be eliminated either by mitophagy (Youle and Narendra, 2011) or autophagy (Barsoum et al., 2006; Twig et al., 2008); these processes prevent the accumulation of damaged mitochondria in cells, thereby preventing the elevation of oxidative stress (Sanderson et al., 2013) and induction of cell apoptosis (Fossati et al., 2016; Xu et al., 2016). In the present study, while high glucose stimulates mitochondrial fission in hippocampal neurons, neither mitophagy proteins nor autophagy proteins are upregulated, suggesting that the elimination processes are not triggered (Ashrafi and Schwarz, 2013). Collectively, the findings provide the explanation for the increased oxidative stress and neuronal apoptosis under high glucose conditions (Fossati et al., 2016; Xu et al., 2016).

Vascular endothelial dysfunction, especially in microvascular, is a critical mechanism underlying diabetic complications (Shi and Vanhoutte, 2017). Endothelial dysfunction in the blood-brain barrier has been intensively studied in diabetic patients and experimental animal models (Mather et al., 2001; Hernandez-Mijares et al., 2013; Daulatzai, 2017; Wang et al., 2017; Bangen et al., 2018; Wang et al., 2020; de Marañón et al., 2021; Jahn et al., 2021; Silva et al., 2021). Treatments with metformin reduce leukocyte-endothelium interaction (Hernandez-Mijares et al., 2013; Wang et al., 2017; de Marañón et al., 2021), inhibit oxygen-derived free radicals (Kapitulnik et al., 2012), improve endothelial-dependent relaxation (Mather et al., 2001), enhance microvascular responsiveness to insulin (Jahn et al., 2021). Thus, it is reasonable to presume that metformin-elicited protective effects on endothelial cells play a partial role in diabetic mice.

In summary, diabetes-induced neuron loss and cognitive impairment are attributed to DRP1 protein activation and increased mitochondrial fragments, leading to enhanced oxidative stress. Metformin inhibits mitochondrial fission, reduces mitochondrial-derived oxidative stress, and restores cognitive function (Figure 7G). Thus, DRP1 may be a potential target for the prevention/management of cognitive impairment in diabetes.

## Data Availability

The original contributions presented in the study are included in the article/Supplementary Material, further inquiries can be directed to the corresponding authors.
