# Supplementary material for: Metformin Protects Against Diabetes-Induced Cognitive Dysfunction by Inhibiting Mitochondrial Fission Protein DRP1
Source: Front Pharmacol. 2022 Mar 22;13:832707. doi: 10.3389/fphar.2022.832707 (PMC8981993; doi:10.3389/fphar.2022.832707)

# HT22

PINK1 Actin

Control Mannitol High-Glucose (15-30-60-100)

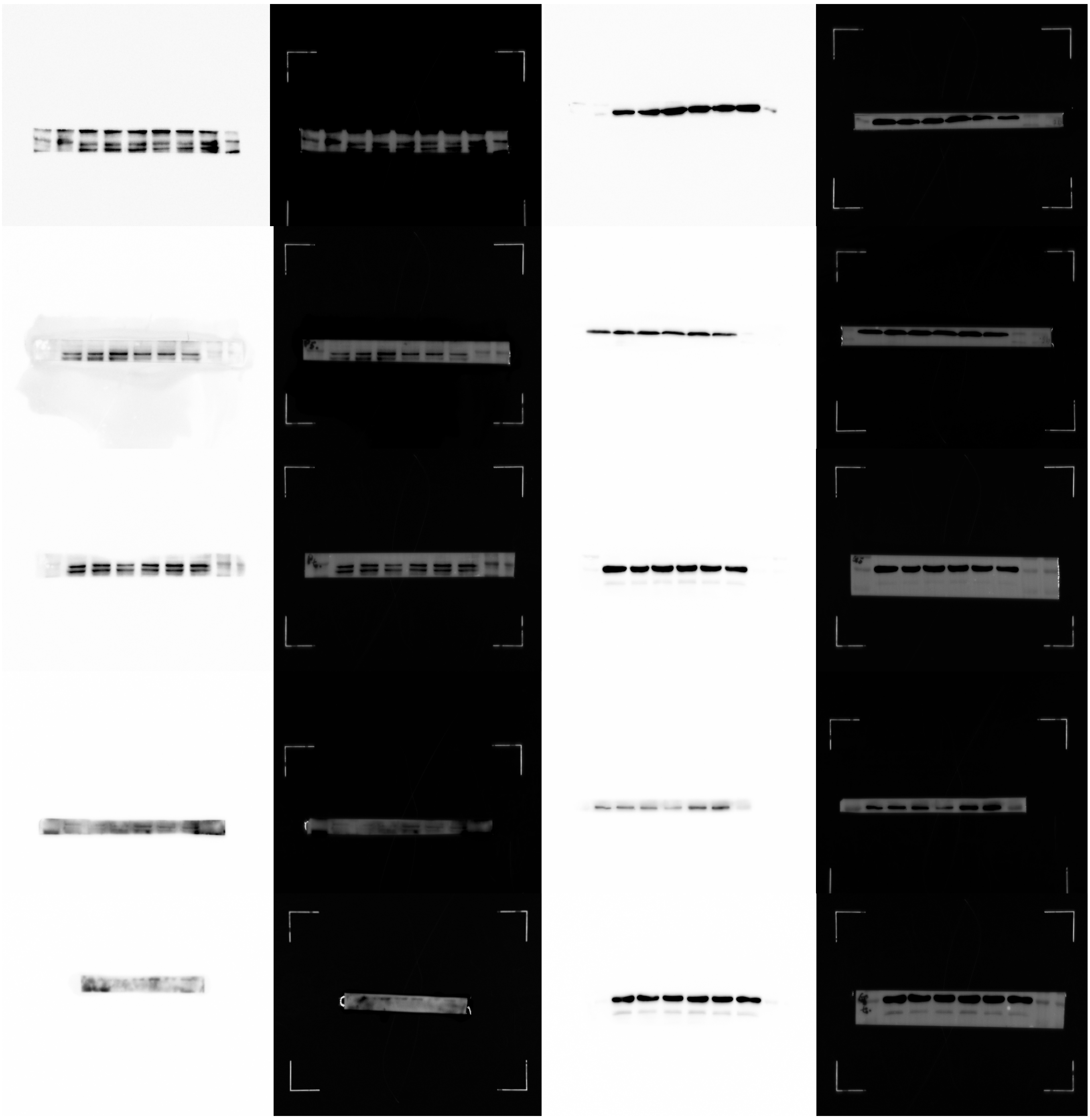

Control Mannitol High-Glucose (15-30-60)

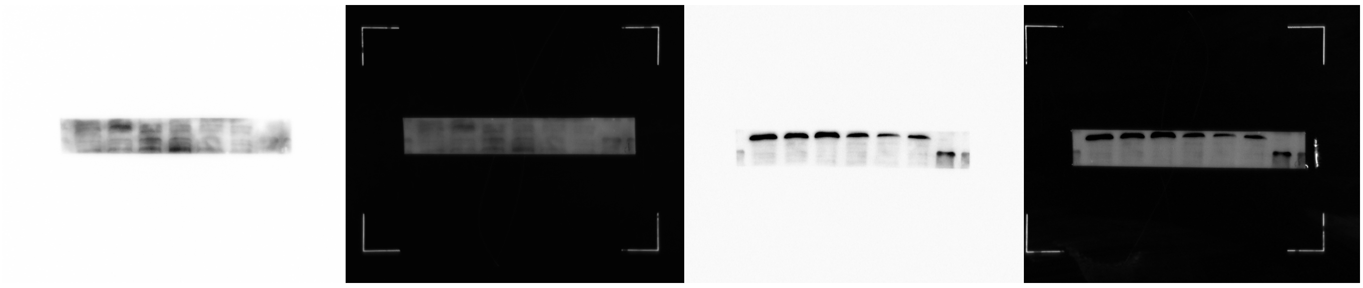

# Neuron

## PINK1 Actin

Control  Mannitol High-Glucose (15-30-60-100)/Control  Mannitol High-Glucose (15-30-60-100)

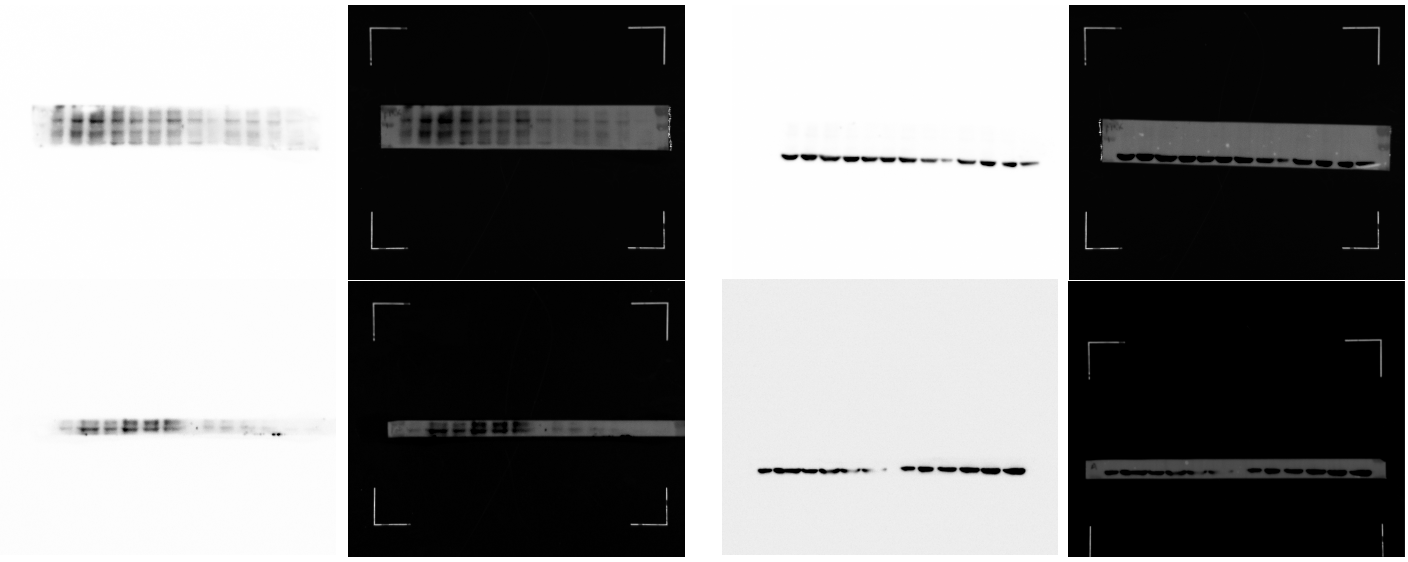

Control Mannitol High-Glucose Metformin Mdivi-1/Control High-Glucose Metformin Mdivi-1(HT22)

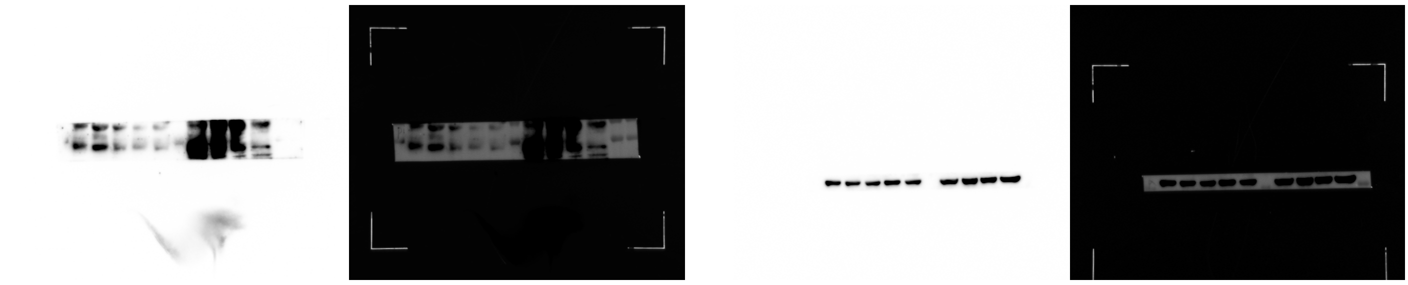

Supplement: Supplementary file 1 [file DataSheet7.PDF]
