# Supplementary material for: Metformin Protects Against Diabetes-Induced Cognitive Dysfunction by Inhibiting Mitochondrial Fission Protein DRP1
Source: Front Pharmacol. 2022 Mar 22;13:832707. doi: 10.3389/fphar.2022.832707 (PMC8981993; doi:10.3389/fphar.2022.832707)

ATG5 Actin

HT22

Control Mannitol High Glucose/Control Mannitol High Glucose/Control Mannitol High Glucose/Control Mannitol High Glucose

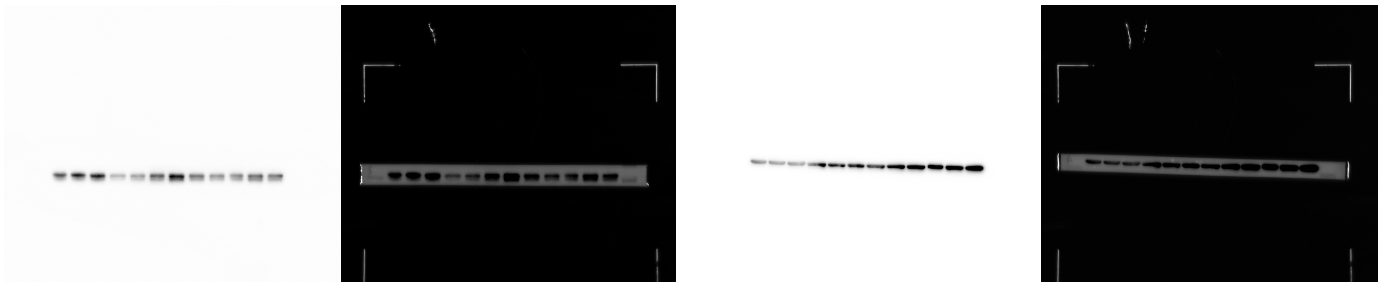

Control Mannitol High-Glucose Metformin Mdivi-1/Control High-Glucose Metformin Mdivi-1(HT22)

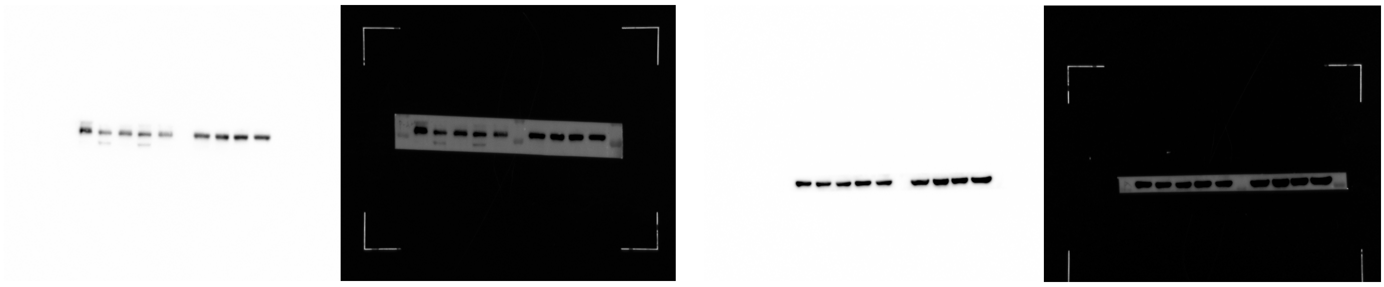

Neuron

Control Mannitol High Glucose/Control Mannitol High Glucose/Control Mannitol High Glucose

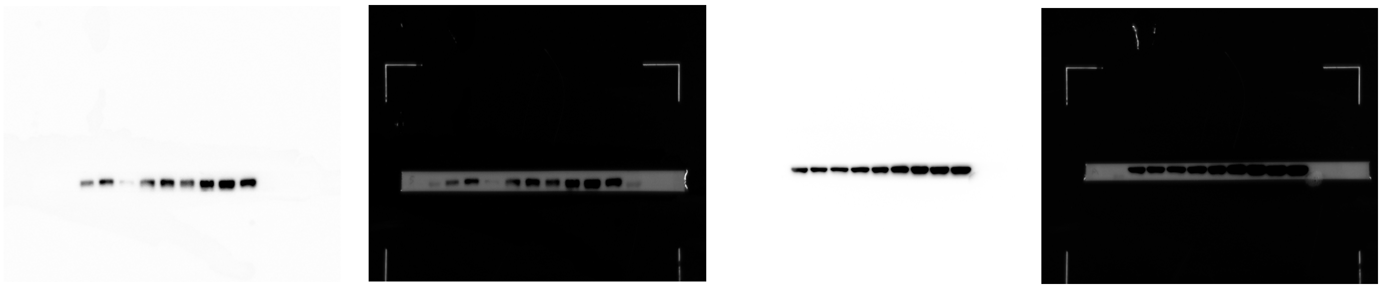

Control Mannitol High Glucose Metformin Mdivi-1/ Control Mannitol High Glucose/ Control Mannitol High Glucose (HT22)

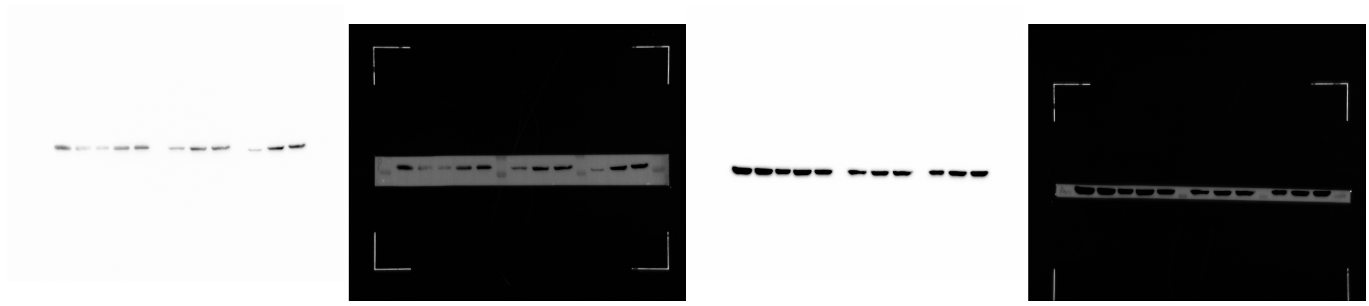

Control Mannitol High Glucose Metformin Mdivi-1

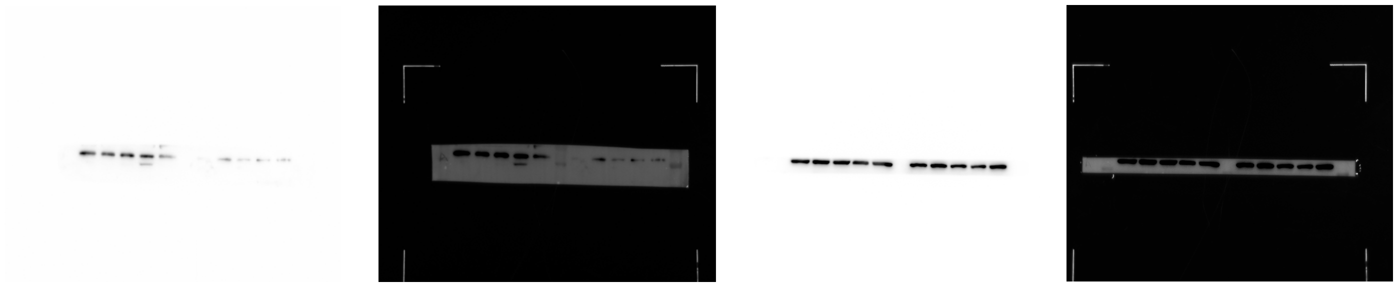

ATG7 Actin

HT22

Control Mannitol High Glucose/Control Mannitol High Glucose/Control Mannitol High Glucose/Control Mannitol High Glucose

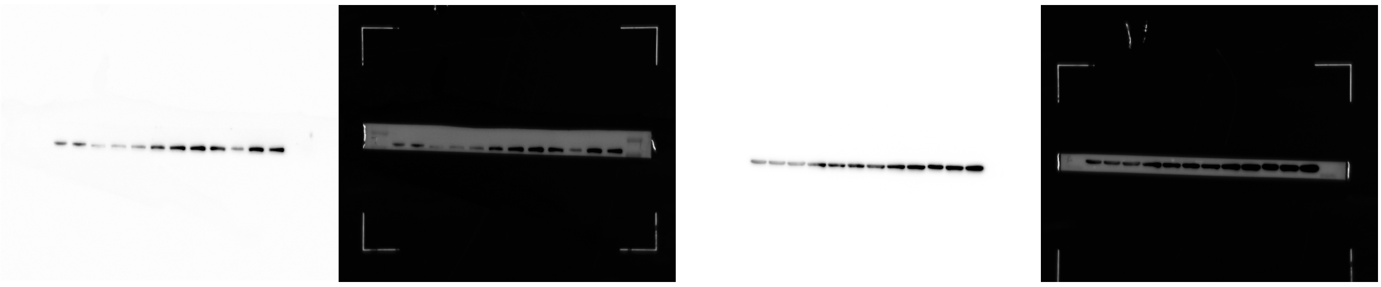

Control Mannitol High-Glucose Metformin Mdivi-1(Neuron)/Control High-Glucose Metformin Mdivi-1(HT22)

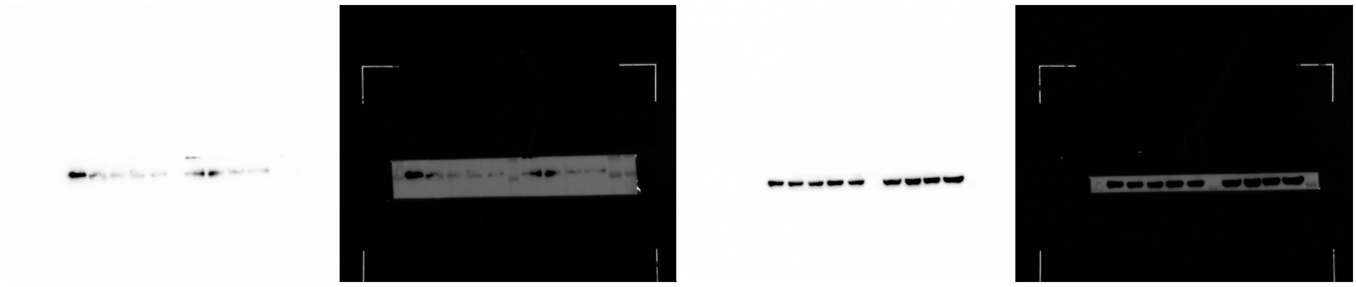

Neuron

Control Mannitol High Glucose/Control Mannitol High Glucose/Control Mannitol High Glucose

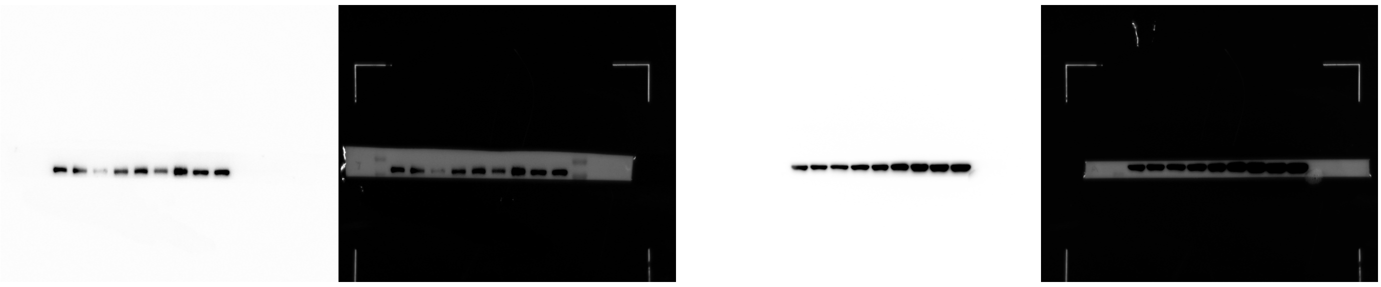

Control Mannitol High Glucose Metformin Mdivi-1/Control Mannitol High Glucose/Control Mannitol High Glucose （HT22）

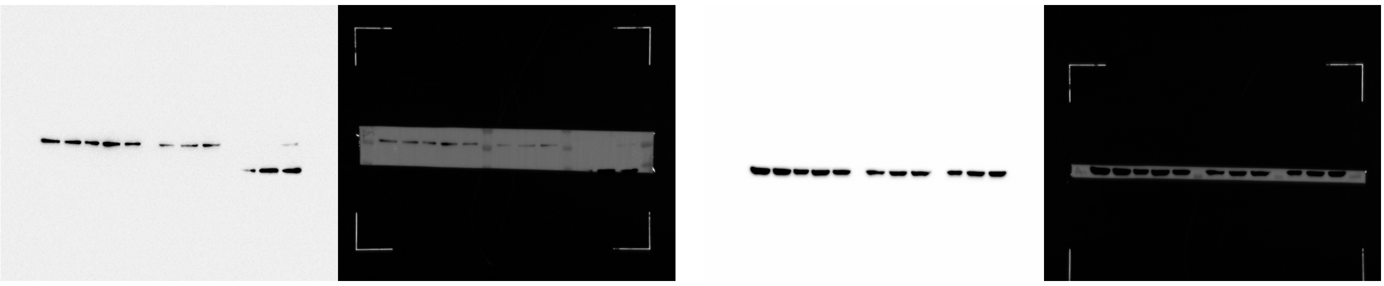

Control Mannitol High Glucose Metformin Mdivi-1

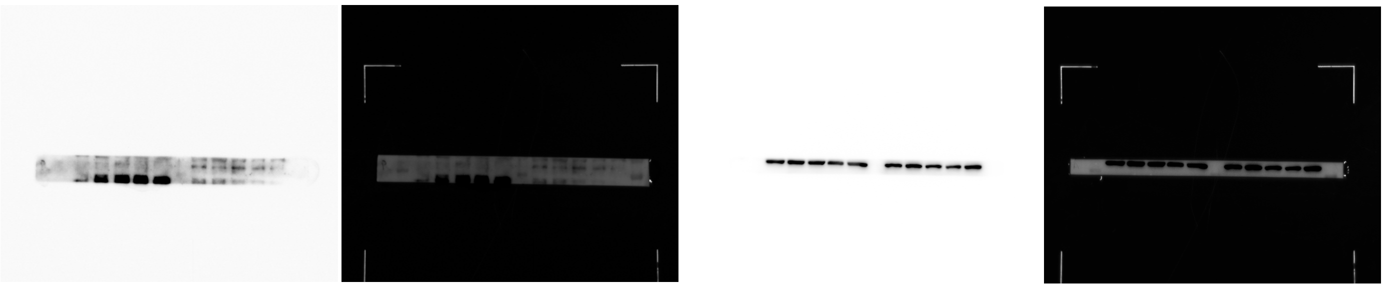

Supplement: Supplementary file 2 [file DataSheet2.PDF]
