# Supplementary material for: Metformin Protects Against Diabetes-Induced Cognitive Dysfunction by Inhibiting Mitochondrial Fission Protein DRP1
Source: Front Pharmacol. 2022 Mar 22;13:832707. doi: 10.3389/fphar.2022.832707 (PMC8981993; doi:10.3389/fphar.2022.832707)

HT22

Control Mannitol High-Glucose (15-30-60-100)/Control Mannitol High-Glucose (15-30-60-100)

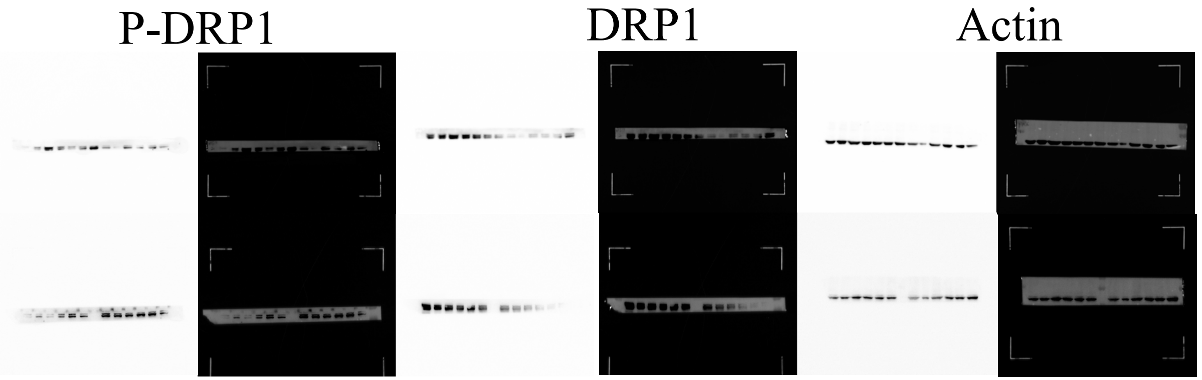

Control Mannitol High-Glucose Metformin Mdivi-1(Neuron)/Control High-Glucose Metformin Mdivi-1

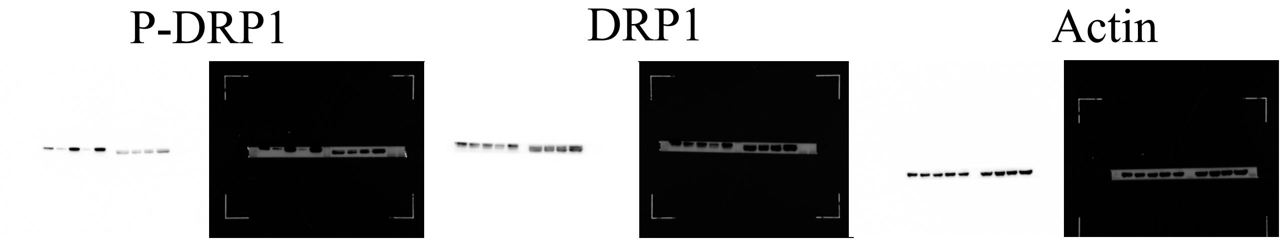

Control Mannitol High-Glucose (15-30-60)/Control Mannitol High-Glucose (15-30-60)

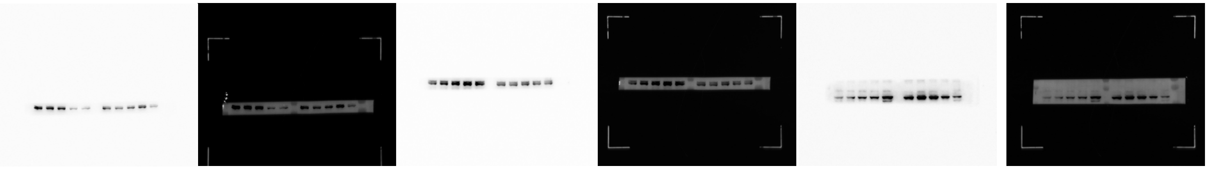

Control High-Glucose Metformin Mdivi-1 GSK621/Control High-Glucose Metformin Mdivi-1 GSK621

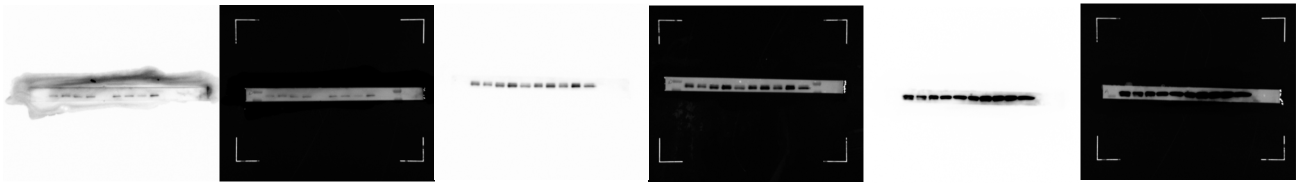

Control High-Glucose Metformin GSK621 Metformin+compound C /
Control High-Glucose Metformin GSK621 Metformin+compound C

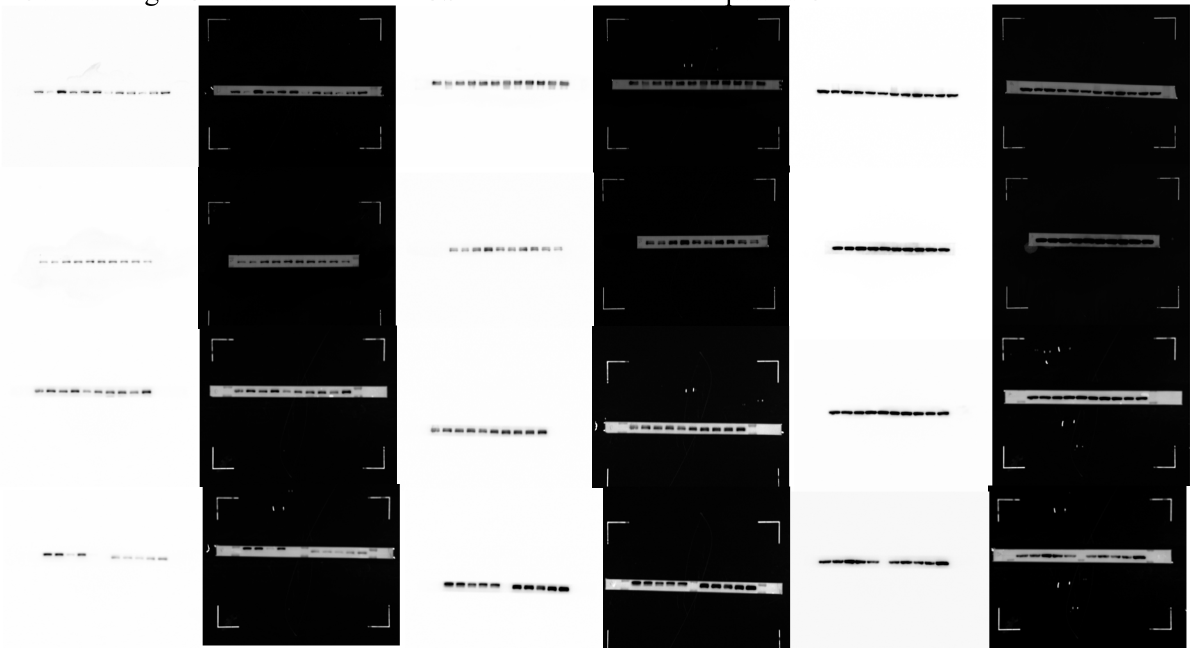

Control Mannitol High-Glucose Metformin Metformin+compound C GSK621 Mdivi-1/
Control High-Glucose Metformin GSK621 Mdivi-1

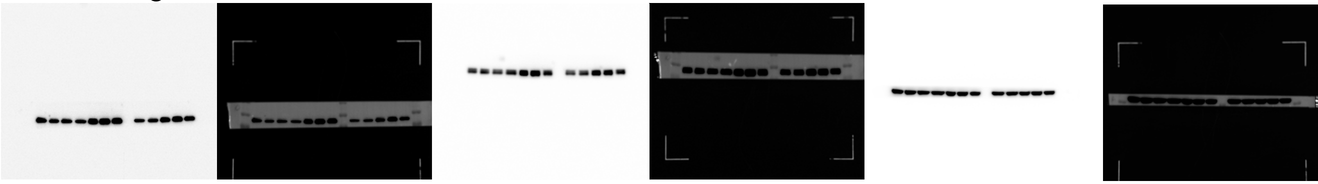

Control Mannitol High-Glucose Metformin Metformin+compound C Mdivi-1/
Control High-Glucose Metformin GSK621 Mdivi-1

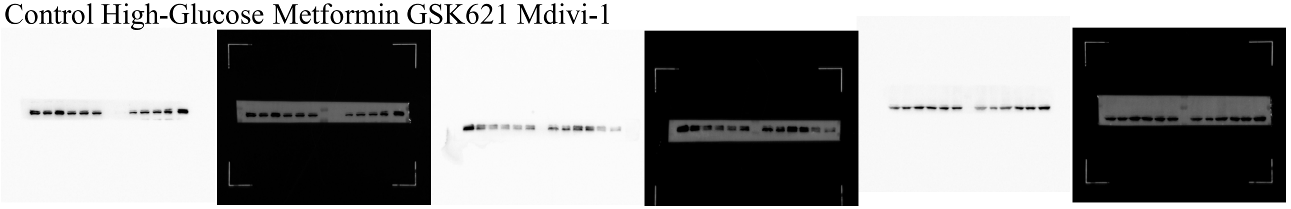

Neuron

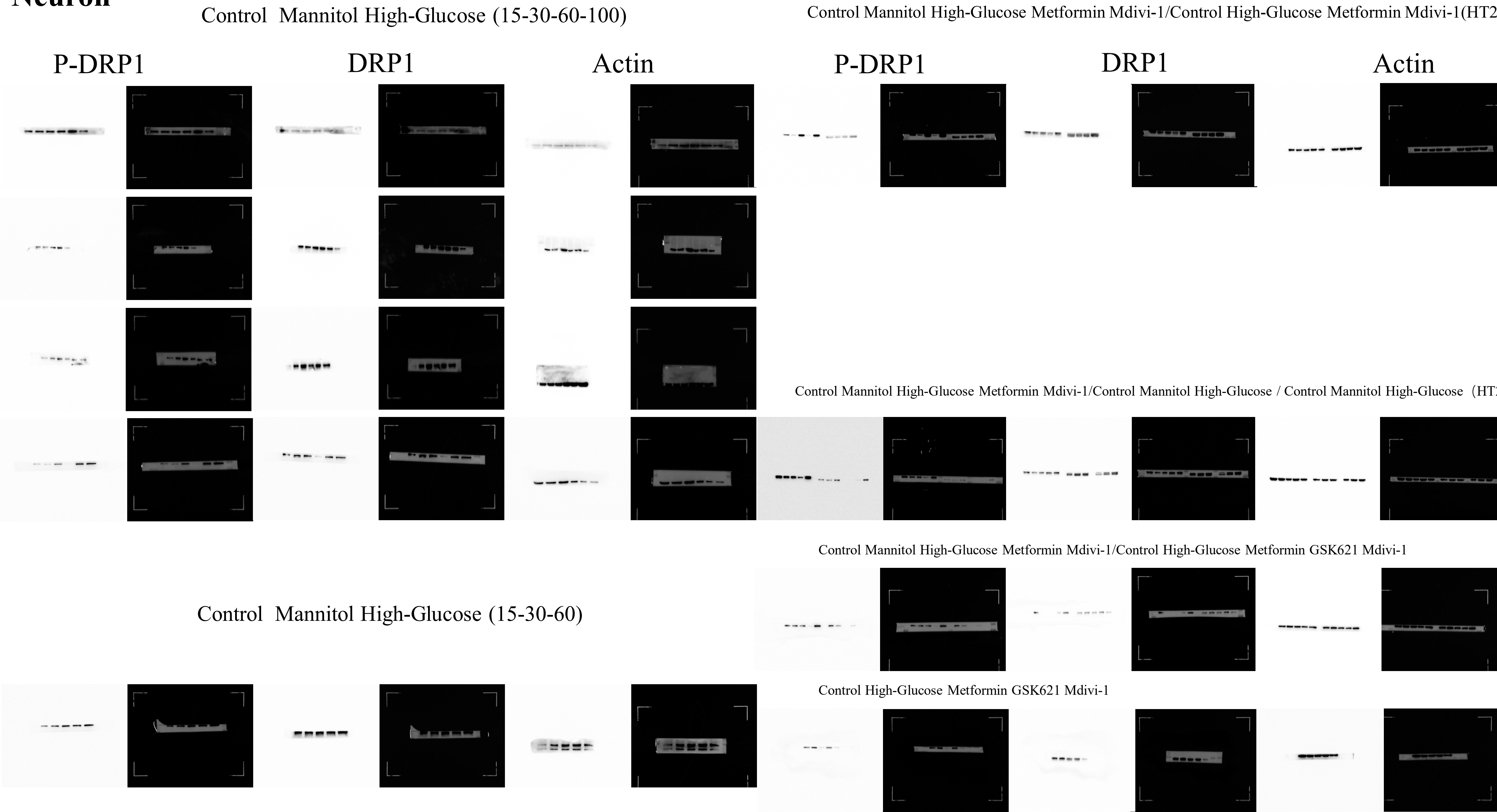

Control Mannitol High-Glucose (15-30-60-100)
Control Mannitol High-Glucose Metformin Mdivi-1/Control High-Glucose Metformin Mdivi-1(HT2
P-DRP1
DRP1
Actin
P-DRP1
DRP1
Actin
Control Mannitol High-Glucose Metformin Mdivi-1/Control Mannitol High-Glucose / Control Mannitol High-Glucose （HT
Control Mannitol High-Glucose Metformin Mdivi-1/Control High-Glucose Metformin GSK621 Mdivi-1
Control Mannitol High-Glucose (15-30-60)
Control High-Glucose Metformin GSK621 Mdivi-1

Neuron

Control High-Glucose Metformin Metformin+compound C Mdivi-1

P-DRP1 DRP1 Actin

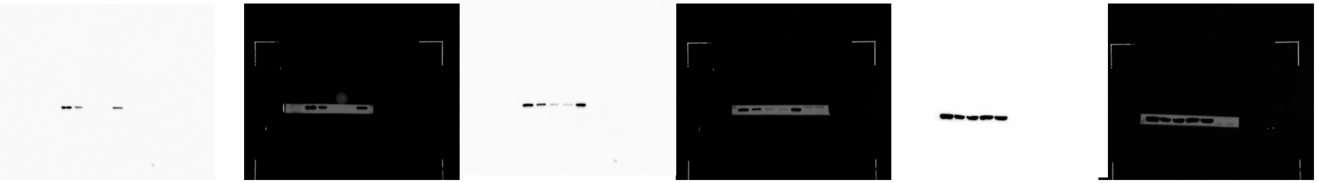

Control High-Glucose Metformin Metformin+compound C GSK621/
Control High-Glucose Metformin Metformin+compound C GSK621

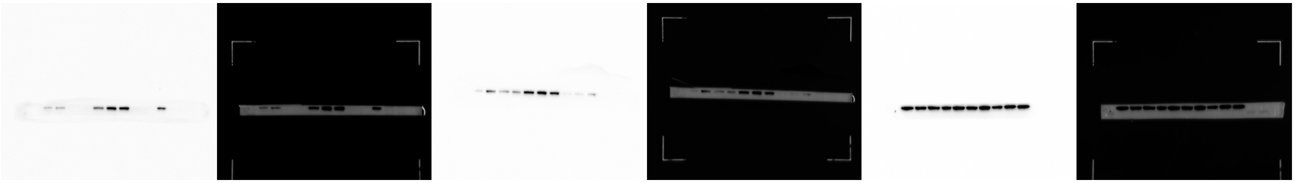

Control High-Glucose Metformin Metformin+compound C Mdivi-1/
Control High-Glucose Metformin Metformin+compound C Mdivi-1

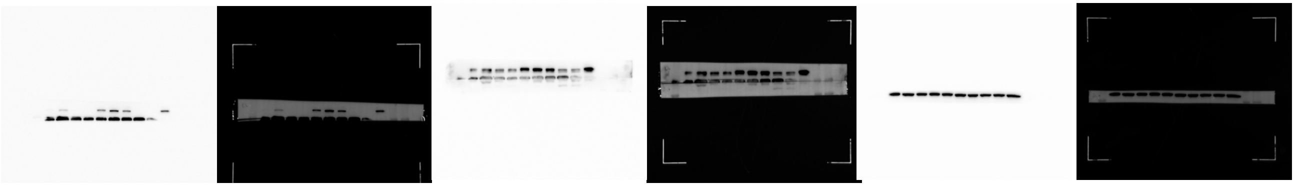

Control Mannitol High-Glucose Metformin Metformin+compound C Mdivi-1 （HT22)/
Control High-Glucose Metformin Metformin+compound C Mdivi-1(Neuron)

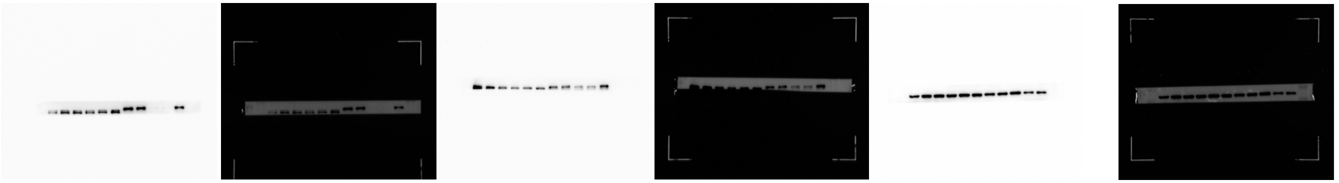

Neuron

Control High-Glucose Metformin GSK621 Metformin+Compound C Mdivi-1

P-DRP1 DRP1 Actin

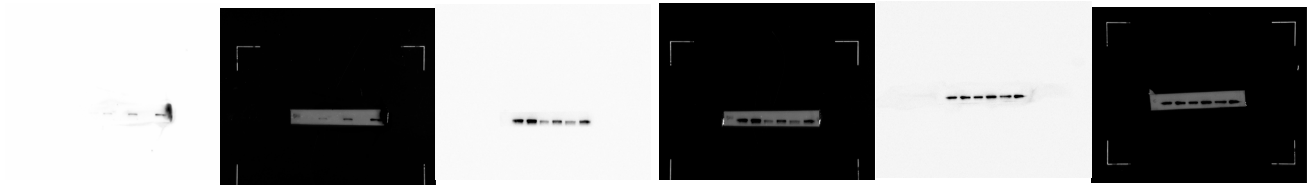

Control High-Glucose Metformin GSK621 Metformin+Compound C Mdivi-1/
Control High-Glucose Metformin GSK621 Metformin+Compound C Mdivi-1

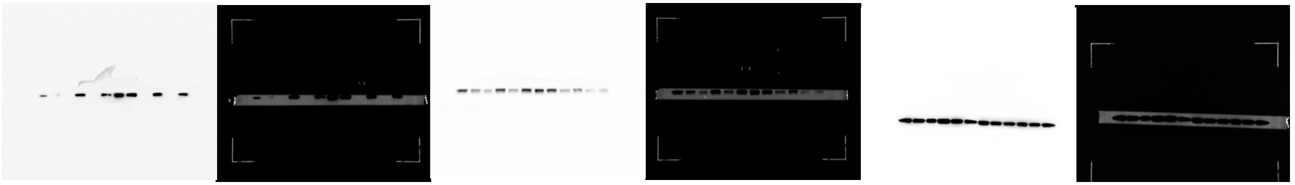

Control High-Glucose Metformin GSK621 Metformin+Compound C /
Control High-Glucose Metformin GSK621 Metformin+Compound C

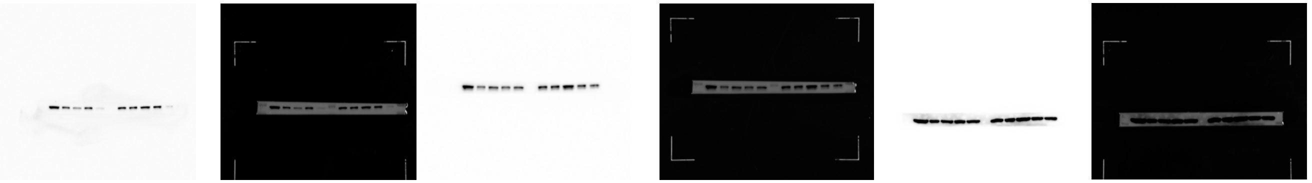

Supplement: Supplementary file 3 [file DataSheet4.PDF]
