# Supplementary material for: Metformin Protects Against Diabetes-Induced Cognitive Dysfunction by Inhibiting Mitochondrial Fission Protein DRP1
Source: Front Pharmacol. 2022 Mar 22;13:832707. doi: 10.3389/fphar.2022.832707 (PMC8981993; doi:10.3389/fphar.2022.832707)

OPA1 Actin

HT22

Control Mannitol High Glucose/Control Mannitol High Glucose/Control Mannitol High Glucose/Control Mannitol High Glucose

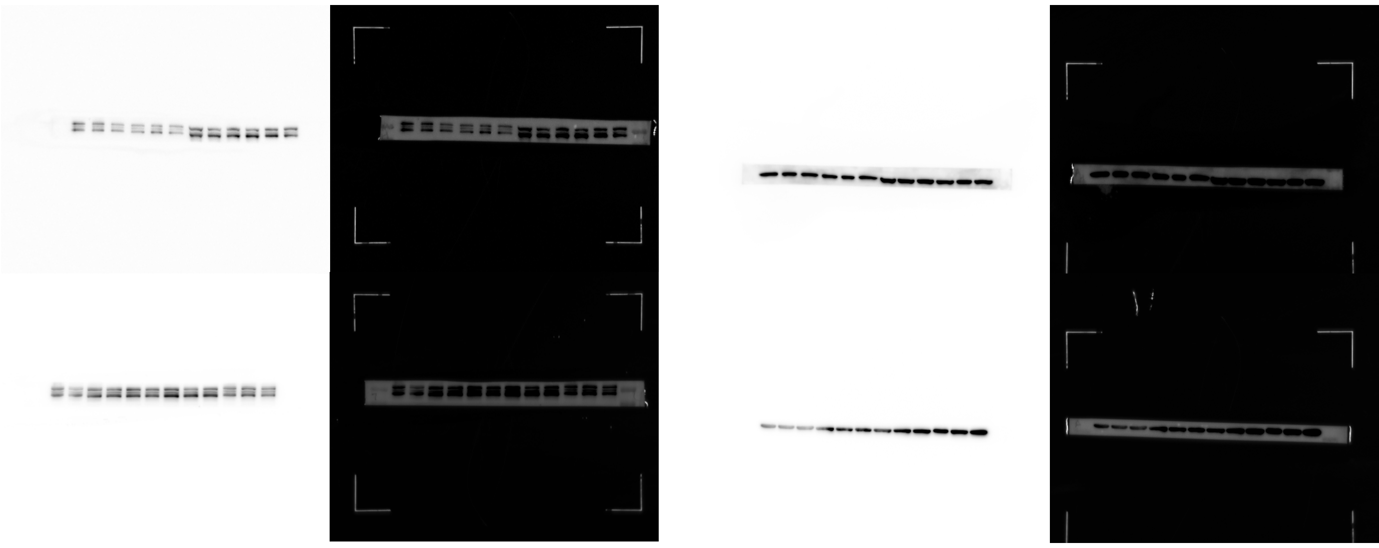

Neuron

Control Mannitol High Glucose/Control Mannitol High Glucose/Control Mannitol High Glucose/Control Mannitol High Glucose

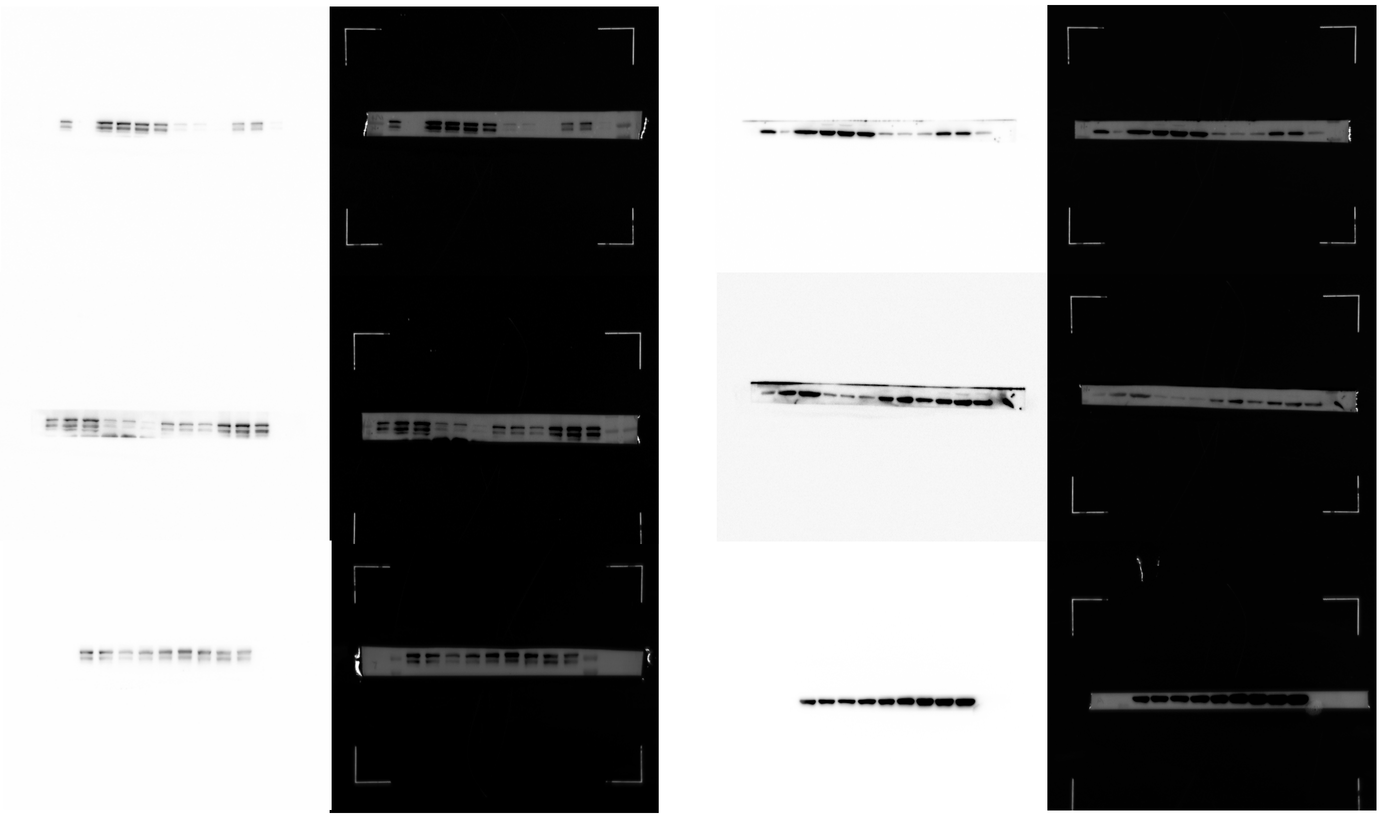

Supplement: Supplementary file 4 [file DataSheet6.PDF]
