# Supplementary material for: Metformin Protects Against Diabetes-Induced Cognitive Dysfunction by Inhibiting Mitochondrial Fission Protein DRP1
Source: Front Pharmacol. 2022 Mar 22;13:832707. doi: 10.3389/fphar.2022.832707 (PMC8981993; doi:10.3389/fphar.2022.832707)
Supplement: Supplementary file 5 [file DataSheet9.pdf]

Metformin protects against diabetes-induced cognitive dysfunction by inhibiting mitochondrial fission protein DRP1

Yan Hu[1,2†], Yile Zhou[1†], Yajie Yang[2], Haihong Tang[2], Yuan Si[3], Zhouyi Chen[1], Yi Shi[3 4*], Hao Fang[1*]

1. Department of Anesthesiology, Zhongshan Hospital, Fudan University, Shanghai, China

2. Department of Anesthesiology, Jinshan Hospital, Fudan University, Shanghai, China

3. Department of Anesthesiology, Minhang Branch, Zhongshan Hospital, Fudan University, Shanghai, China

4. Institute of Clinical Science, Zhongshan Hospital, Fudan University, Shanghai, China

5. Shanghai Key Laboratory of Organ Transplantation, Zhongshan Hospital, Fudan University, Shanghai, China

†These authors have contributed equally to this work

Corresponding Author

Professor Fang, Hao

Department of Anesthesiology, Zhongshan Hospital, Fudan University, Shanghai, China

E-mail: drfanghao@163.com

Dr. Shi, Yi

Institute of Clinical Science, Zhongshan Hospital, Fudan University, Shanghai, China

Shanghai Key Laboratory of Organ Transplantation, Zhongshan Hospital, Fudan

University, Shanghai, China

E-mail: shi.yi@zs-hospital.sh.cn

ORCID: 0000-0003-3005-9655

Supplementary figure 1

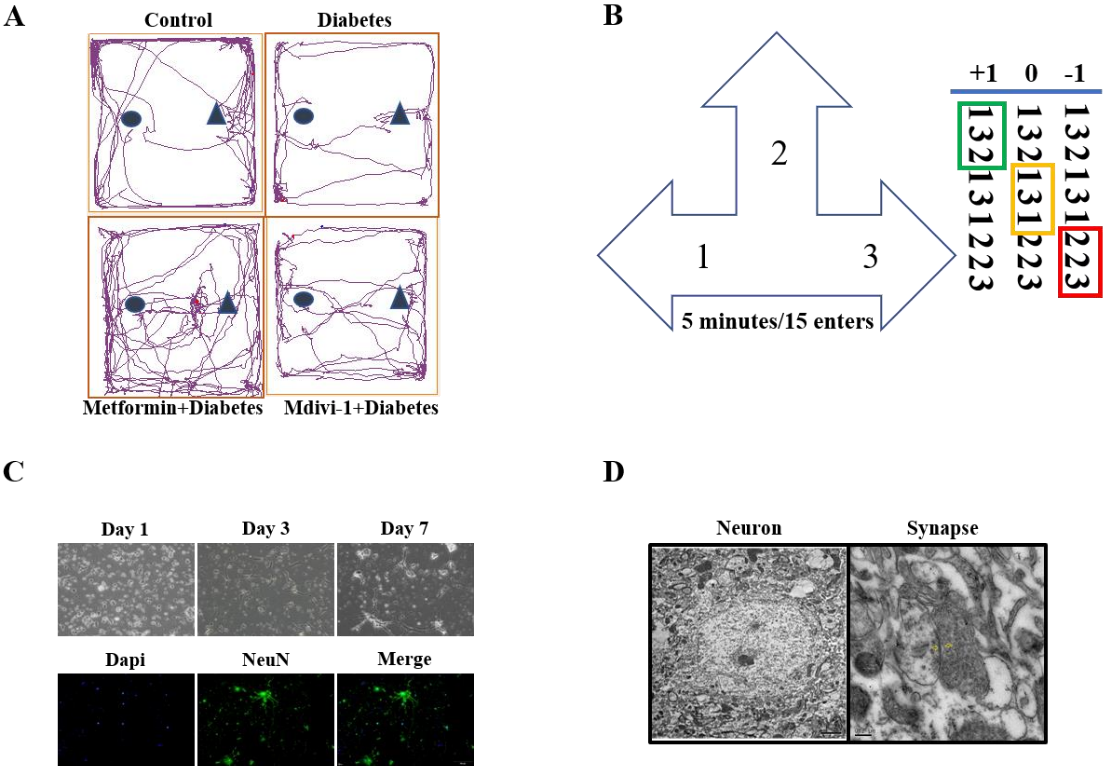

Supplementary Figure 1

Supplementary Figure 1 (A) the path of mice in novel object recognition test, the triangle represents the new object and the circle represents the old object (B) Protocol and point count in T-maze test; (C) Identification of hippocampus primary neuron by NeuN staining (200×); (D) TEM of neuron (5000×) and synapse (50000×)

Supplementary Figure 2

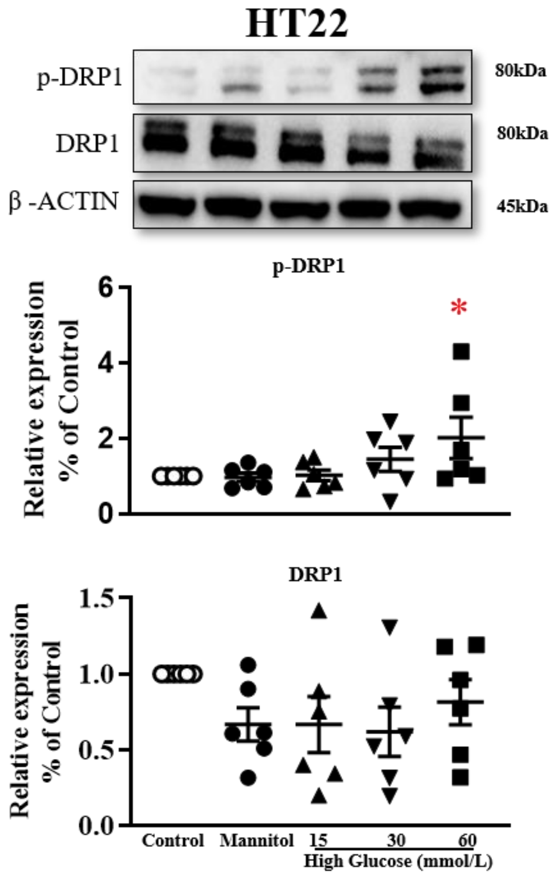

Supplementary Figure 2

Supplementary Figure 2 In HT22 cells, six-hour high glucose stimulation induces a higher phosphorylated level of DRP1 at serine 616 residue, but not the total protein, in a dose-dependent manner. Mannitol (60 mM) incubation does not affect DRP1 expression in cultured cells

Supplementary Figure 3

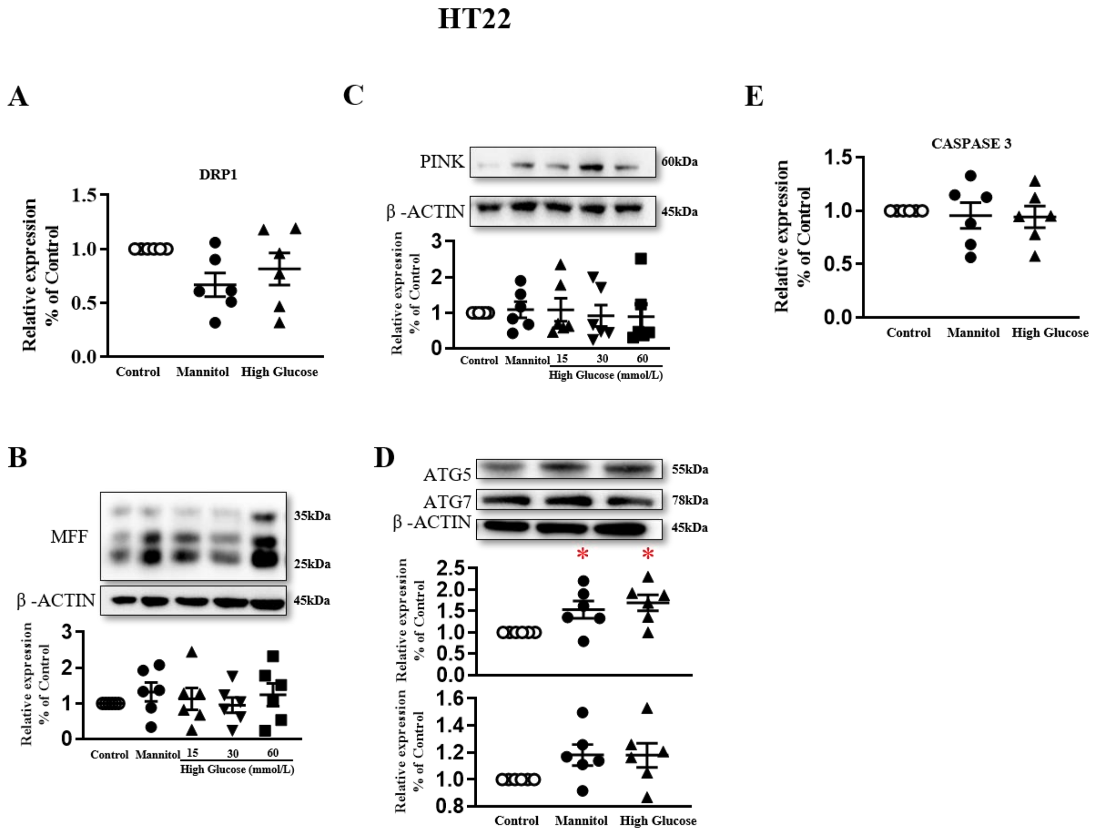

Supplementary Figure 3

Supplementary Figure 3 In HT22, (A)Densitometric quantification of total DRP1; (B) Representative blots (upper) and densitometric quantification (lower) of MFF; (C) Representative blots (upper) and densitometric quantification (lower) of PINK, (D) ATG5 and ATG7; (E) Densitometric quantification of total caspase 3

Supplementary Figure 4

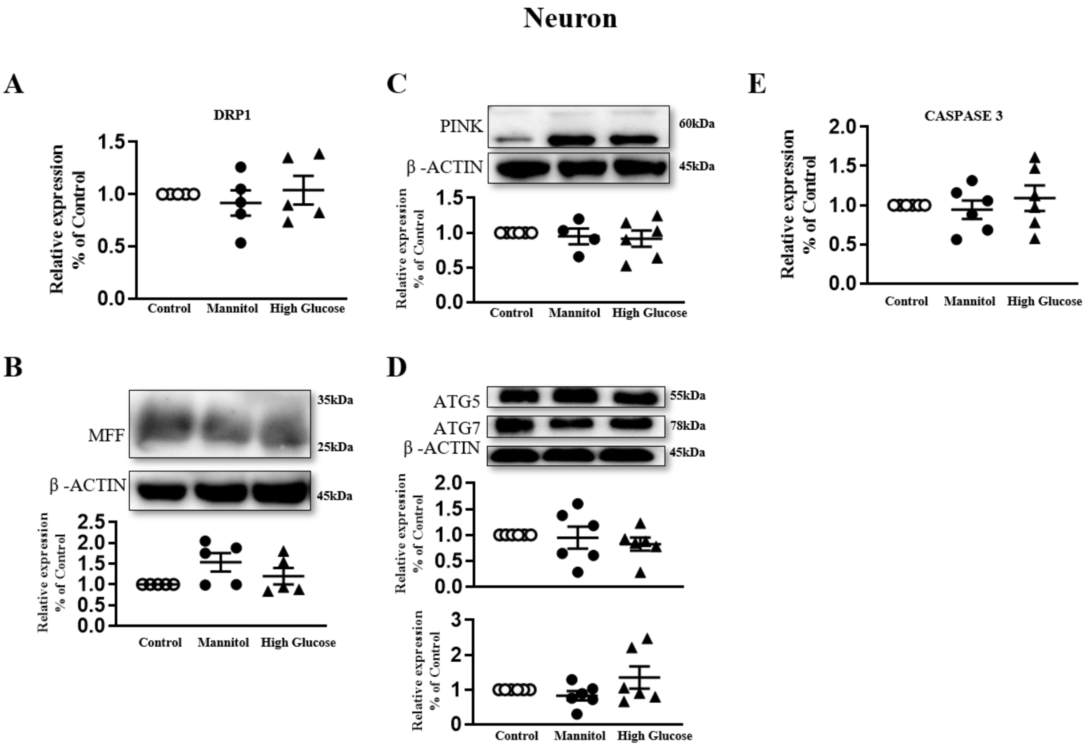

Supplementary Figure 4

Supplementary Figure 4 In primary neuron, (A) Densitometric quantification of total DRP1; (B) Representative blots (upper) and densitometric quantification (lower) of MFF, (C) PINK, (D) ATG5 and ATG7; (E) Densitometric quantification of total caspase 3

Supplementary Figure 5

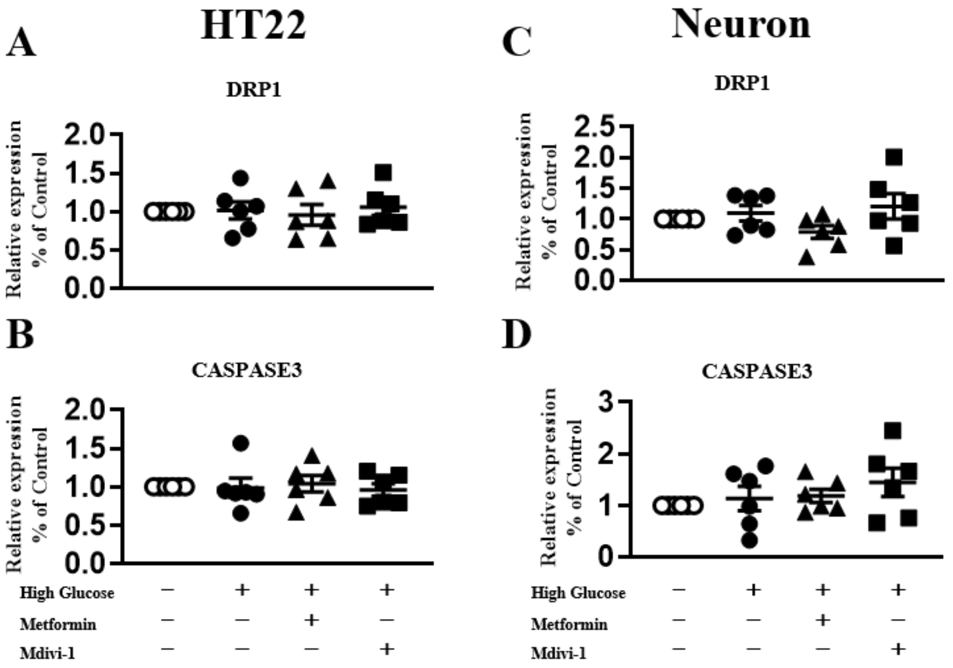

Supplementary Figure 5

Supplementary Figure 5 Densitometric quantification of total DRP1(A, C) and caspase 3 (B, D) in HT22 (A and B) and primary neuron (C and D) with metformin or Mdivi-1 incubation.

Supplementary Figure 6

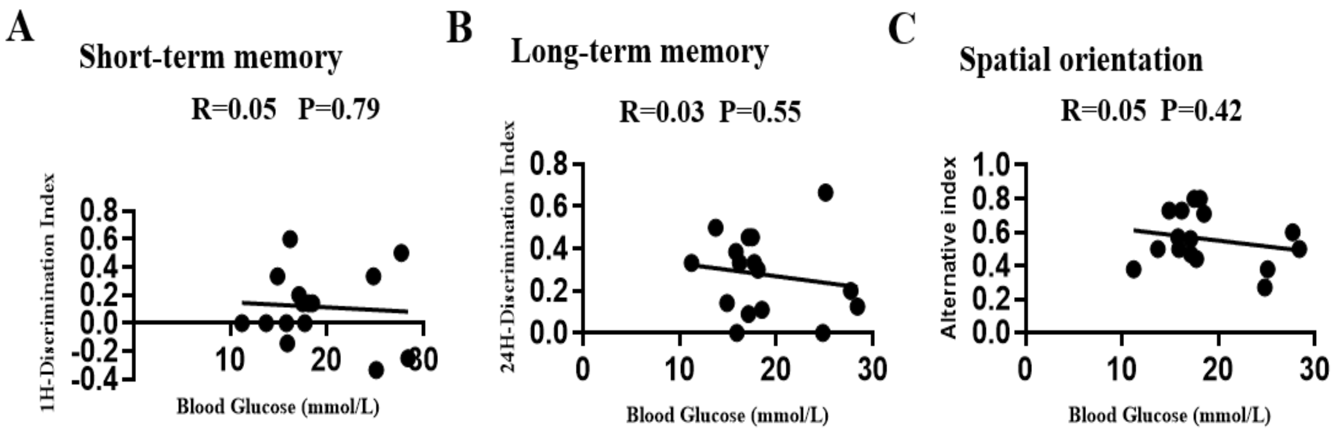

Supplementary Figure 6

Supplementary Figure 6 Correlation of serum glucose and mice cognitive performance. (A) short-term memory (B) long-term memory (C) spatial orientation
