# Supplementary material for: Metformin Protects Against Diabetes-Induced Cognitive Dysfunction by Inhibiting Mitochondrial Fission Protein DRP1
Source: Front Pharmacol. 2022 Mar 22;13:832707. doi: 10.3389/fphar.2022.832707 (PMC8981993; doi:10.3389/fphar.2022.832707)

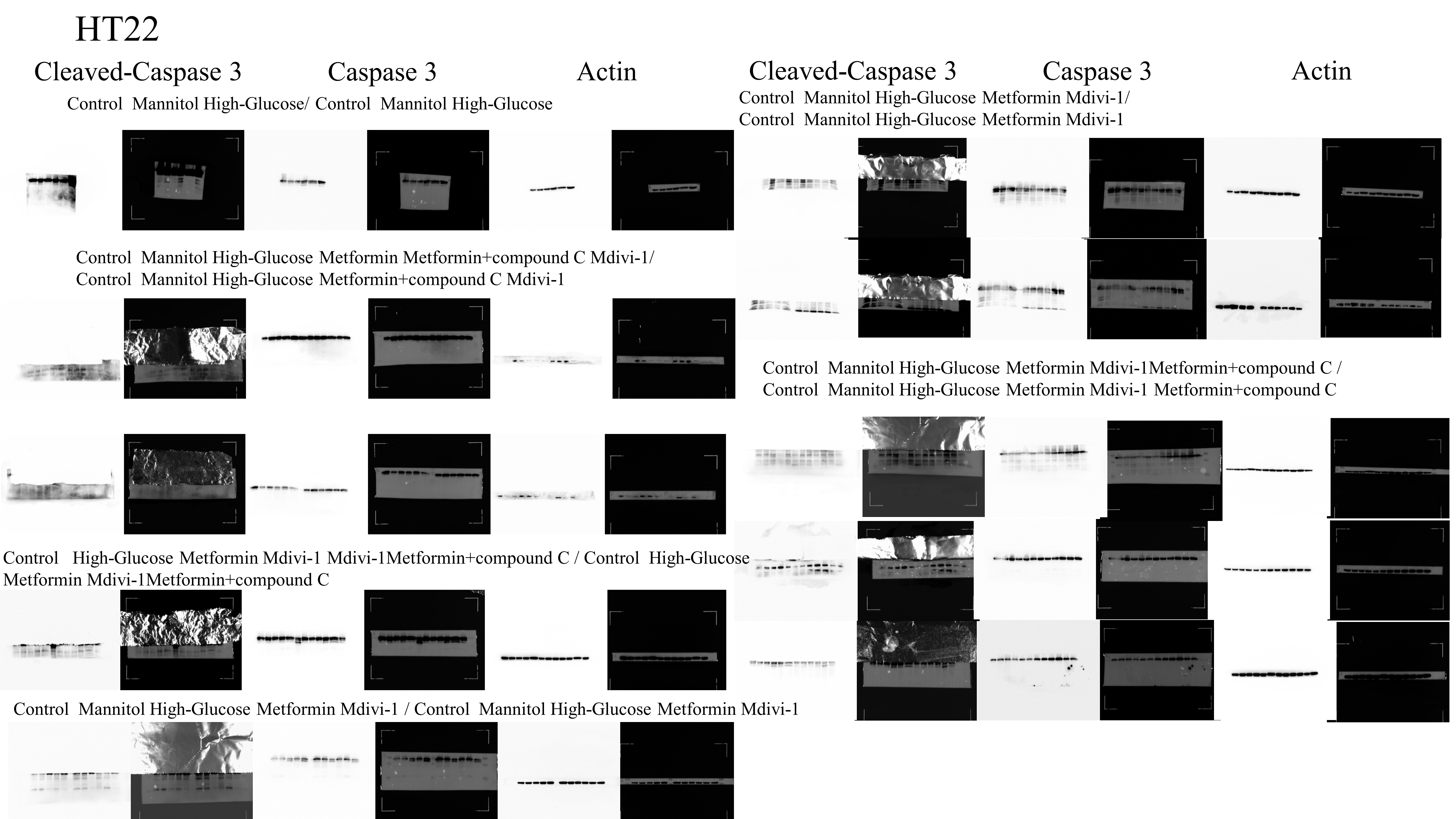

HT22
Cleaved-Caspase 3
Caspase 3
Actin
Cleaved-Caspase 3
Caspase 3
Actin
Control Mannitol High-Glucose/ Control Mannitol High-Glucose
Control Mannitol High-Glucose Metformin Mdivi-1/
Control Mannitol High-Glucose Metformin Mdivi-1
Control Mannitol High-Glucose Metformin Metformin+compound C Mdivi-1/
Control Mannitol High-Glucose Metformin+compound C Mdivi-1
Control Mannitol High-Glucose Metformin Mdivi-1Metformin+compound C /
Control Mannitol High-Glucose Metformin Mdivi-1 Metformin+compound C
Control High-Glucose Metformin Mdivi-1 Mdivi-1Metformin+compound C / Control High-Glucose
Metformin Mdivi-1Metformin+compound C
Control Mannitol High-Glucose Metformin Mdivi-1 / Control Mannitol High-Glucose Metformin Mdivi-1

Neuron

Cleaved-Caspase 3 Caspase 3 Actin

Control Mannitol High-Glucose (15-30-60)

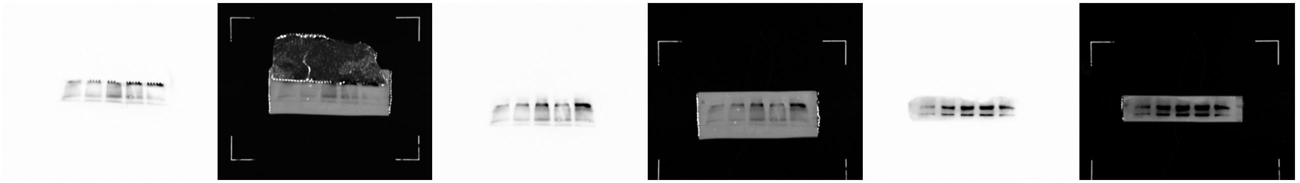

Cleaved-Caspase 3+Caspase 3 Actin

Control High-Glucose Metformin Metformin+compound C Mdivi-1

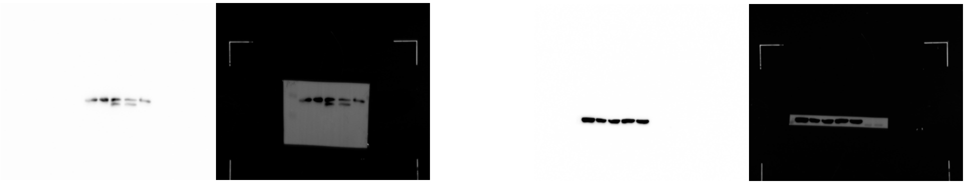

Control Mannitol High-Glucose Metformin Mdivi-1/Control High-Glucose Metformin Mdivi-1(HT22)

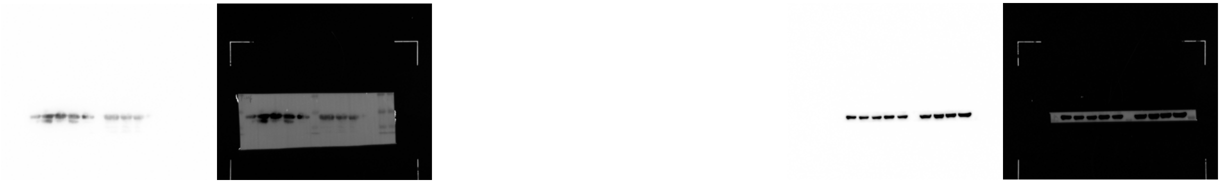

Control High-Glucose Metformin GSK621 Mdivi-1

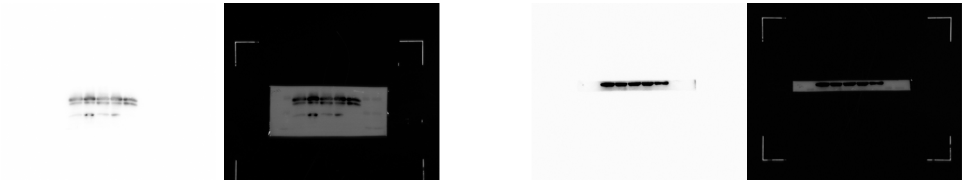

Control High-Glucose Metformin Mdivi-1 GSK621 Metformin+compound C /
Control High-Glucose Metformin Mdivi-1 GSK621 Metformin+compound C

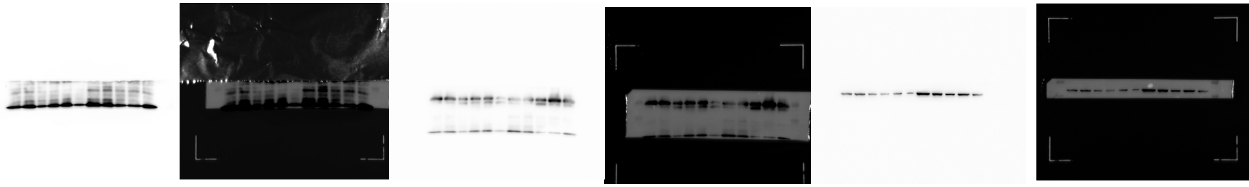

Control Mannitol High-Glucose Metformin Mdivi-1/Control High-Glucose Metformin GSK621 Mdivi-1

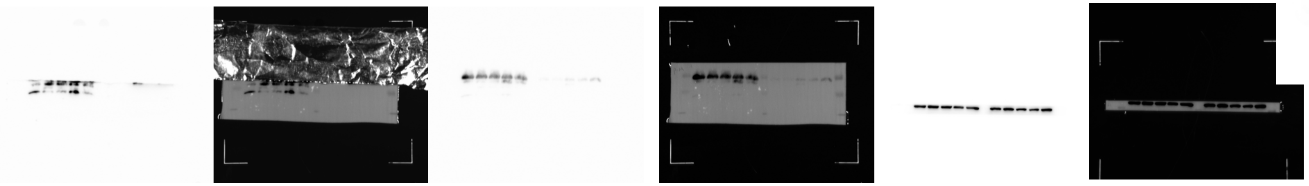

Control High-Glucose Metformin GSK621 Metformin+Compound C Mdivi-1

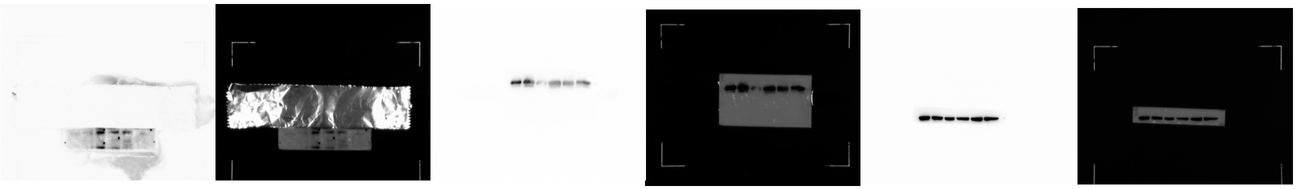

Supplement: Supplementary file 6 [file DataSheet3.PDF]
