# Supplementary material for: Metformin Protects Against Diabetes-Induced Cognitive Dysfunction by Inhibiting Mitochondrial Fission Protein DRP1
Source: Front Pharmacol. 2022 Mar 22;13:832707. doi: 10.3389/fphar.2022.832707 (PMC8981993; doi:10.3389/fphar.2022.832707)

HT22

P-AMPK AMPK Actin

Control High-Glucose Metformin GSK621 Metformin+compound C /
Control High-Glucose Metformin GSK621 Metformin+compound C

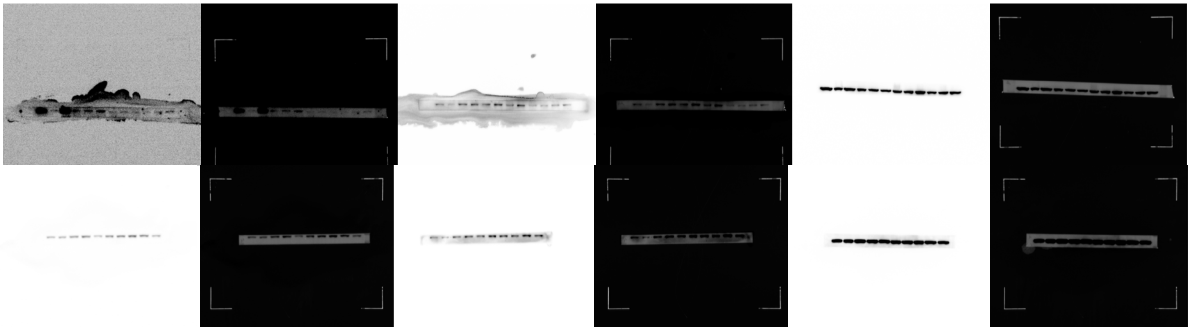

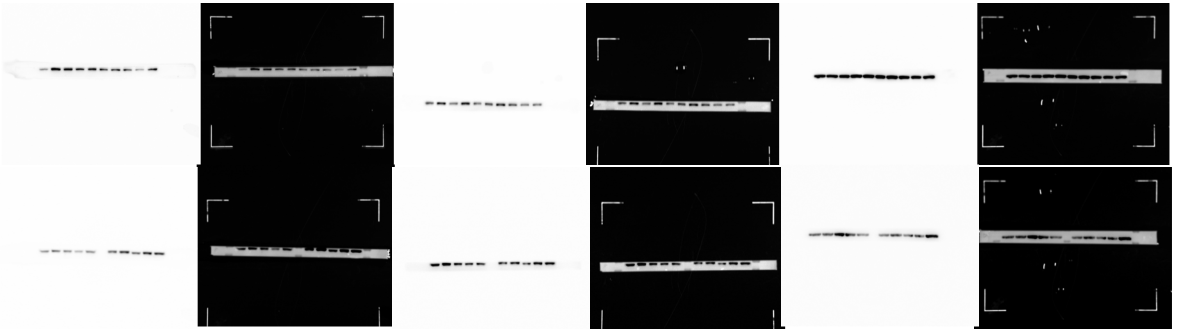

Control High-Glucose Metformin GSK621 Metformin+Compound C Mdivi-1/
Control High-Glucose Metformin GSK621 Metformin+Compound C Mdivi-1

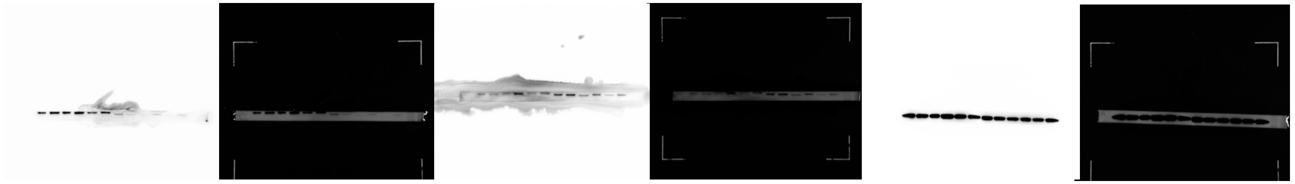

P-AMPK AMPK Actin

Control High-Glucose Metformin Mdivi-1 GSK621/Control High-Glucose Metformin Mdivi-1 GSK621

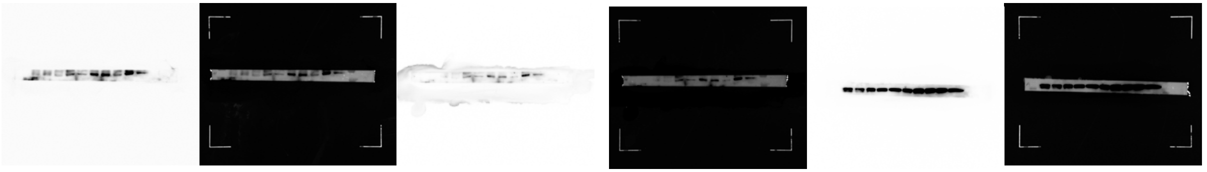

Control Mannitol High-Glucose Metformin Metformin+compound C Mdivi-1 （HT22)/
Control High-Glucose Metformin Metformin+compound C Mdivi-1(Neuron)

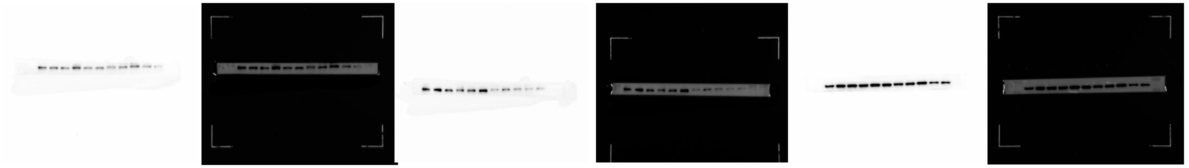

Control High-Glucose Metformin Metformin GSK621

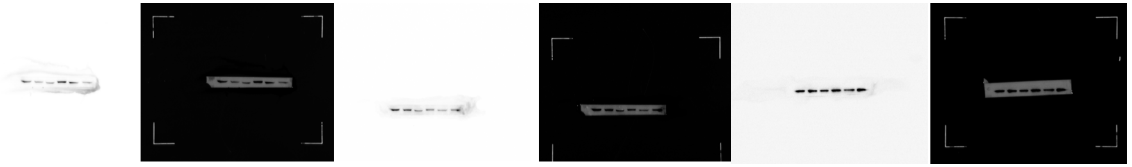

Neuron

Control Mannitol High-Glucose Metformin Mdivi-1/Control High-Glucose Metformin Mdivi-1

P-AMPK AMPK Actin

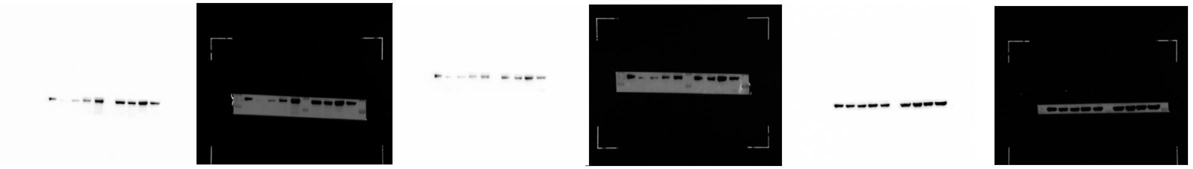

Control Mannitol High-Glucose Metformin Mdivi-1

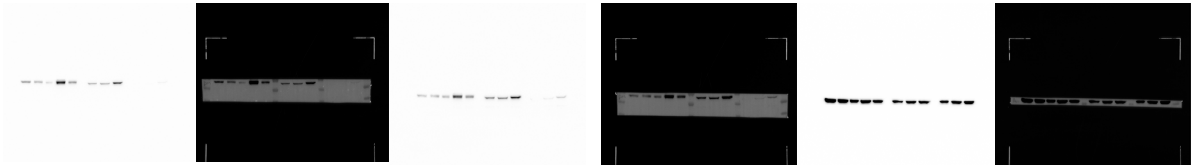

Control High-Glucose Metformin GSK621 Mdivi-1

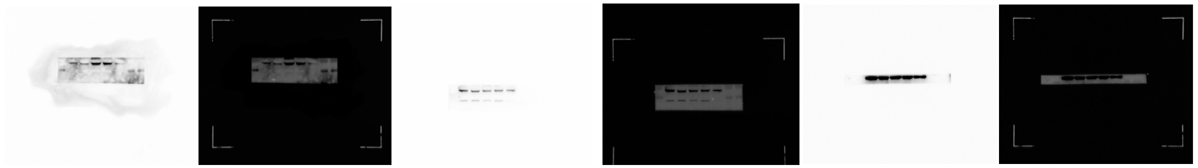

Control Mannitol High-Glucose Metformin Metformin+compound C Mdivi-1 （HT22)/
Control High-Glucose Metformin Metformin+compound C Mdivi-1(Neuron)

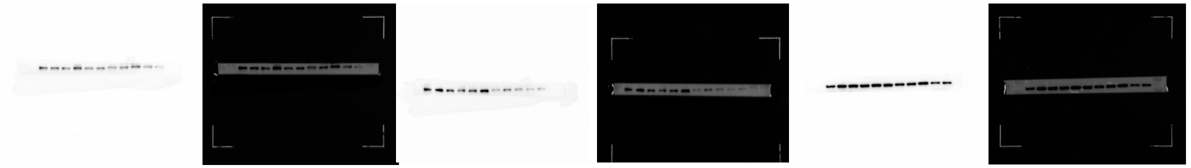

Control High-Glucose Metformin GSK621 Metformin+Compound C /
Control High-Glucose Metformin GSK621 Metformin+Compound C

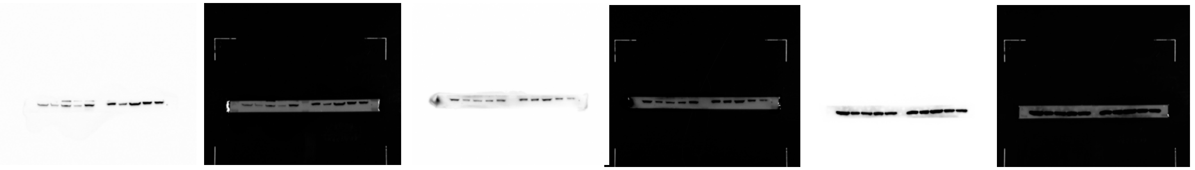

Control High-Glucose Metformin Metformin+compound C Mdivi-1 GSK621/
Control High-Glucose Metformin Metformin+compound C Mdivi-1

P-AMPK AMPK Actin

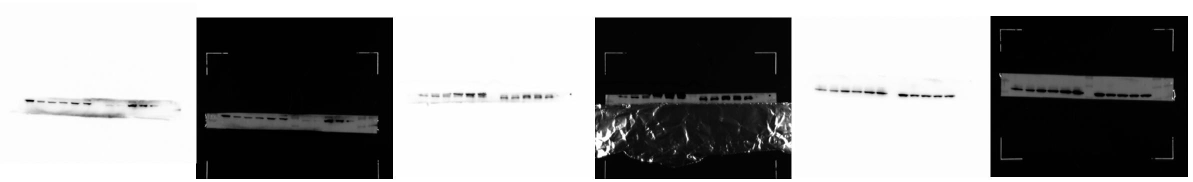

Control High-Glucose Metformin Metformin+compound C Mdivi-1

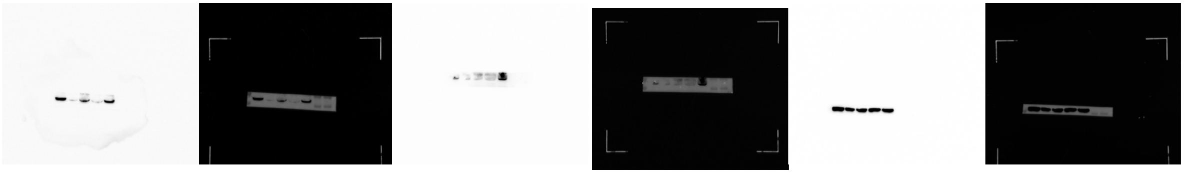

Control High-Glucose Metformin Metformin+compound C GSK621/
Control High-Glucose Metformin Metformin+compound C GSK621

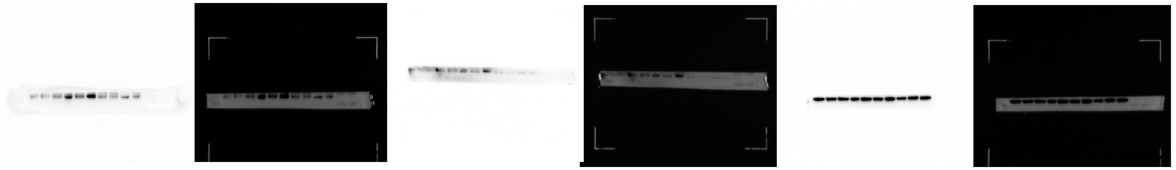

Control High-Glucose Metformin Metformin+compound C Mdivi-1/
Control High-Glucose Metformin Metformin+compound C Mdivi-1

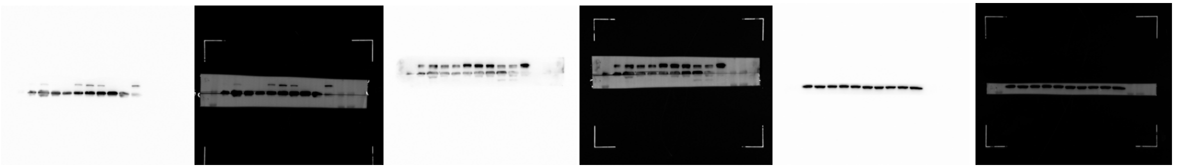

Supplement: Supplementary file 7 [file DataSheet1.PDF]
