# Supplementary material for: Metformin Protects Against Diabetes-Induced Cognitive Dysfunction by Inhibiting Mitochondrial Fission Protein DRP1
Source: Front Pharmacol. 2022 Mar 22;13:832707. doi: 10.3389/fphar.2022.832707 (PMC8981993; doi:10.3389/fphar.2022.832707)
Supplement: Supplementary file 9 [file DataSheet8.PDF]

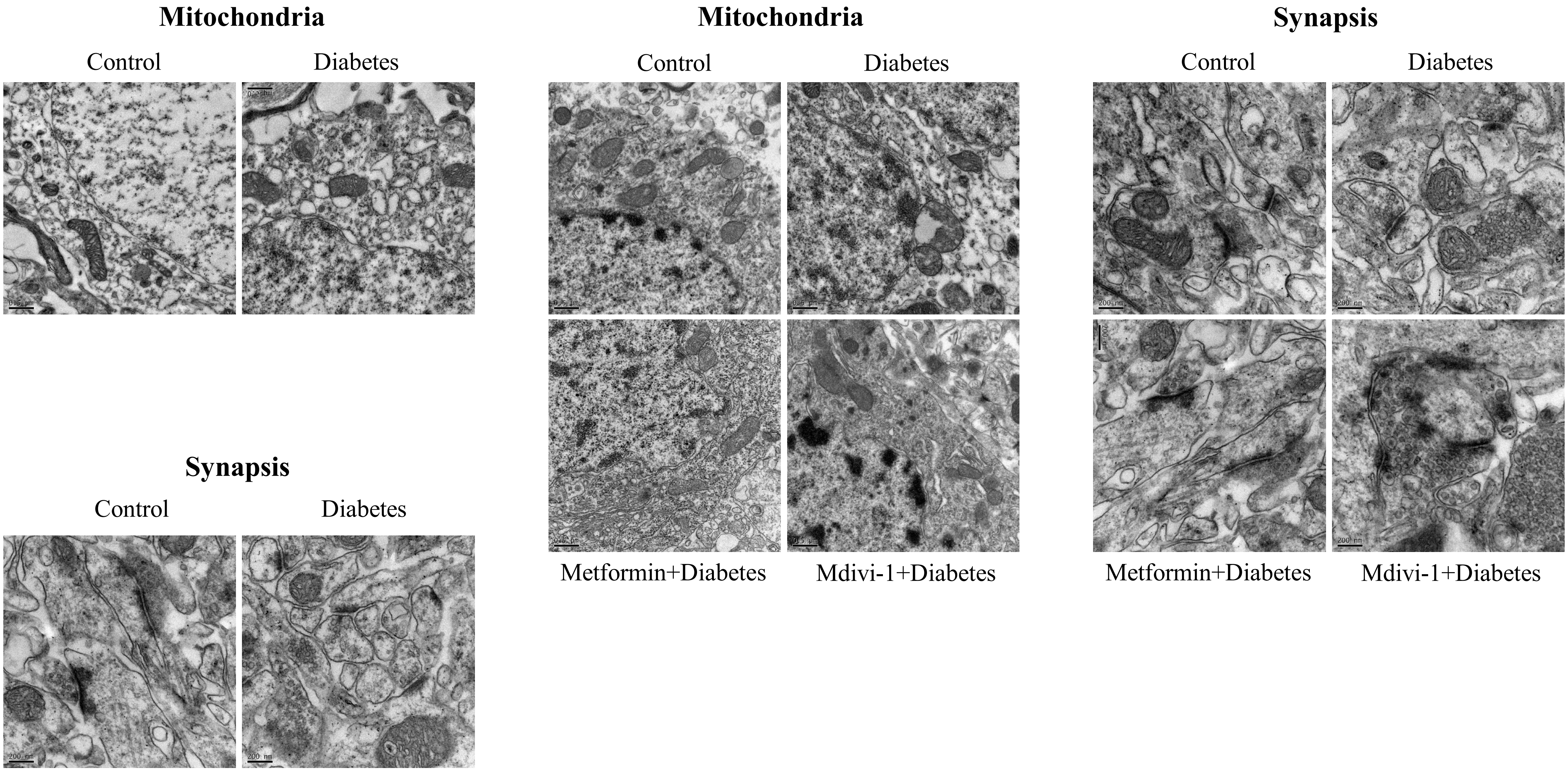
Mitochondria
Control
Diabetes
Synapsis
Control
Diabetes
Mitochondria
Control
Diabetes
Metformin+Diabetes
Mdivi-1+Diabetes
Synapsis
Control
Diabetes
Metformin+Diabetes
Mdivi-1+Diabetes
